# The gastrointestinal microbiome in dairy cattle is constrained by the deterministic driver of the region and the modified effect of diet

**DOI:** 10.1186/s40168-022-01453-2

**Published:** 2023-01-20

**Authors:** Limei Lin, Zheng Lai, Jiyou Zhang, Weiyun Zhu, Shengyong Mao

**Affiliations:** 1 Laboratory of Gastrointestinal Microbiology, Jiangsu Key Laboratory of Gastrointestinal Nutrition and Animal Health, National Center for International Research On Animal Gut Nutrition, College of Animal Science and Technology, Nanjing Agricultural University, Nanjing, 210095 China; 2 Ruminant Nutrition and Feed Engineering Technology Research Center, College of Animal Science and Technology, Nanjing Agricultural University, Nanjing, 210095 China

**Keywords:** Dairy cattle, Gastrointestinal microbiome, Segmental heterogeneity, Dietary regimes, Carbon handoffs, Interspecies hydrogen transfer

## Abstract

**Background:**

Dairy cattle (*Bos taurus*), especially Holstein cows, which are the highest-producing dairy animals and are widely bred to provide milk products to humans, rely critically on their associated gastrointestinal tract (GIT) microbiota to digest plant feed. However, the region-specific taxonomic composition and function of the GIT microbiome in dairy cattle and the mechanistic basis for the diet-induced effects remain to be elucidated.

**Results:**

We collected 120 digesta samples from 10 GIT regions of 12 Holstein cows fed forage- and grain-based diets and characterized their GIT microbiome via functional shotgun metagenomics and the resolution of metagenome-assembled genomes. Our results demonstrated that the GIT microbiome was mainly partitioned into three distinct clusters, four-chambered stomach, small intestine, and large intestine. Moreover, we found that the four-chambered stomach microbiome with the highest diversity had a strong ability to degrade recalcitrant polysaccharide substrates, underpinned by the prevalence of potential cellulosome-­producing and plant-derived polysaccharide utilization loci-encoding consortia. In contrast, the post-gastric intestinal microbiome orchestrated alternative fermentation pathways to adapt to nutrient availability and energy acquisition. Diet shifts selectively modified the metabolic cascades of the microbiome in specific GIT regions, evidenced by the loss of fiber-degrading taxa and increased hydrogen sinks in propionate after grain introduction.

**Conclusions:**

Our findings provide new insights into GIT microbial organization and function in dairy cattle by GIT regions and diet regimes, which offers clues for improving animal production and health in the future.

Video Abstract

**Supplementary Information:**

The online version contains supplementary material available at 10.1186/s40168-022-01453-2.

## Background

Ruminants are crucial partners in human society, providing important sources of nutritious foods such as milk and meat from nonhuman-edible plant biomass [1]. Such conversion occurs mainly from microbes residing in the gastrointestinal tract (GIT) via alimentary and endogenous trophic systems. This process is accompanied by the output of greenhouse gases that cause global climate change, such as methane [2], and the loss of gross dietary energy that occurs during enteric methanogenesis, which leads to a significant reduction in feed efficiency [3]. Therefore, the GIT microbiome has been recognized as an important factor in feed efficiency and environmental impacts. Characterizing the features of the GIT microbiome in ruminants is essential for improving feed efficiency and reducing the environmental impact.

Dairy cattle are bred for their ability to produce large volumes of milk, particularly Holstein cows, which are the world’s highest-production dairy animals. For decades, researchers have extensively examined the importance of the microbiome in the rumen, an important organ for digestion, fermentation, and nutrition in dairy cattle. Recently, there has been an increasing recognition that the microbiome in the post-gastric intestine also contributes greatly to production efficiency and animal health [3, 4]. However, our knowledge about microbial communities remains limited. The GIT is an anatomically multi-region system. The oxygen levels, pH, and nutrient availability differ across regions [5], such as the nutrient-rich forestomach and nutrient-scarce hindgut. These physiological factors may lead to significant segmental differences in the microbial composition and function of dairy cattle.

We have previously characterized the GIT bacterial community of dairy cattle via 16S rRNA gene sequencing [6]. However, the functional heterogeneity of the GIT microbiome remains to be explored with a higher microbial resolution. In addition, the knowledge gap regarding spatial trophic systems, such as the metabolic handoff and competitive utilization of carbon and hydrogen among different functional groups, impedes a profound understanding of the microbial community in the small and large intestines. Metagenomic sequencing, particularly metagenome-assembled genomes (MAGs), has provided in-depth insight into previously unknown taxa and functional pathways [4, 7]. Therefore, a genome-resolved understanding is urgently needed to investigate microbial organization and function in the GIT regions and the biological mechanisms of the region-specific trophic systems of dairy cattle.

Dietary intervention alters the composition of nutrient substrates. Consequently, the GIT microbiome has new metabolic characteristics to adapt to the changed lumen substrates [8, 9]. In most countries worldwide, dairy cattle are generally fed forage- and grain-based diets, which differ in their fiber and starch content [10]. To date, no studies have explored the diet-induced dynamics of the entire GIT microbiome in dairy cattle. In this regard, efforts to elucidate whether diet regimes can reshape the segmental effects on the GIT microbiota of dairy cattle and how region-specific microbes modify their degradative and fermentation capabilities through competitive utilization to meet diet shifts are important.

Here, we constructed a microbial gene catalog and MAGs from 120 GIT metagenomic samples of 12 Holstein cows fed forage- and grain-based diets. These integrated data enabled us to (1) phylogenetically resolve and define region-specific “enterotypes”; (2) reveal region-specific substrate preferences and distinct fermentation signatures, and assign functions to specific taxa from the proximal to distal GIT; (3) decipher how specific microbial phylotypes process region-specific carbon handoffs and hydrogen sinks; and (4) elucidate the diet-induced modification of carbon metabolic processes and competitive hydrogen utilization by specific taxa. Taken together, our findings reveal the region-specific trophic system and diet-driven metabolic flexibility of the GIT microbiome in dairy cattle to adapt to nutrient availability and energy acquisition. Our data also reveals the relative contribution of region versus diet regimes in shaping the structure and function of the gastrointestinal microbiome in dairy cattle.

## Results

### Constructing a GIT microbial gene catalog in dairy cattle

We collected 120 content samples covering 10 GIT regions from 12 dairy cattle fed forage- and grain-based diets. Over 2.7 terabytes (Tb) of metagenomic data was obtained with an average of 22.8 gigabytes (Gb) per sample, totaling 18.2 billion sequencing reads with a length of 150 bp (Additional file 1: Table S1). Following the removal of host and diet DNA contamination, 1.6 Tb of the GIT microbial data remained. We then obtained 90.7 million contigs and 153 million open reading frames (ORFs) via metagenomic assembly and ORF prediction (see “Methods” section). After clustering at 95% nucleotide sequence identity, we generated a non-redundant microbial gene catalog of 45,886,195 genes (average length 607 bp). According to the currently available databases, only half of the genes (48.8%, 22,398,032) were taxonomically classified, of which, 97.7% could be assigned to bacteria, and the remaining genes were classified as archaeal (0.58%), eukaryotic (0.30%), and viral species (0.17%). We also found that 61.4% (28,170,604), 62.3% (28,574,080), and 3.7% (1,692,7920) of genes were annotated as clusters of orthologous groups of proteins, KEGG orthologous groups (KOs), and carbohydrate-active enzymes (CAZymes), respectively. This gene catalog represents a comprehensive GIT microbiome of dairy cattle and was used in subsequent studies.

### Microbial composition landscape along the GIT of dairy cattle

**Fig. 1:**
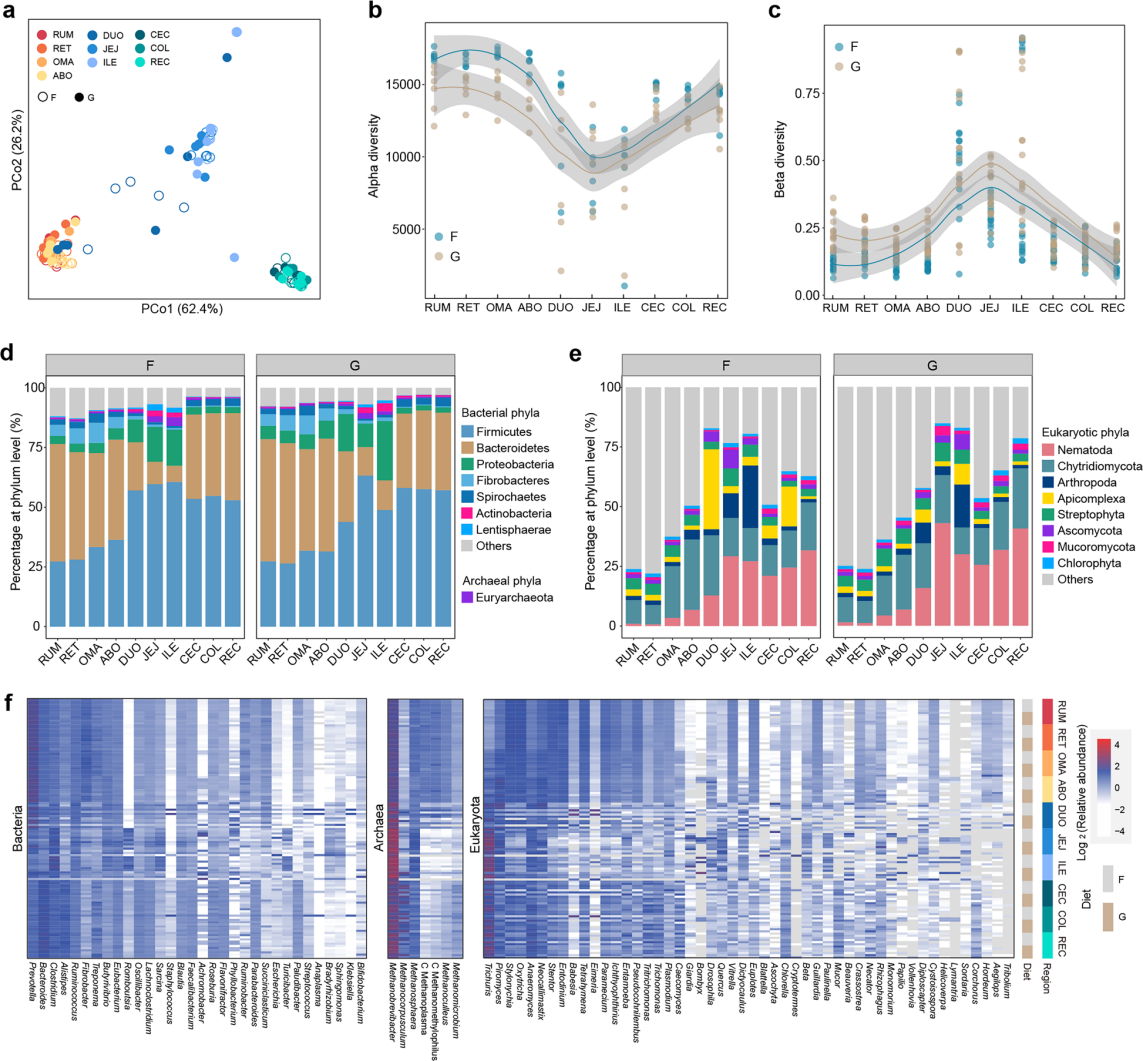
Region-specific distribution of distinct microbial populations from the proximal to distal gastrointestinal tract (GIT) of dairy cattle in the forage-based (F) and grain-based (G) diets, respectively. **a** Principal coordinate analysis (PCoA) profile among the 10 GIT regions. Alpha diversity (**b** richness index) and beta diversity (**c** Bray–Curtis) across the GIT regions. **d** Relative abundance (%) of dominant phyla, including the bacterial, archaeal, and eukaryotic (**e**) communities from the proximal to distal GIT. **f** Relative abundance of major genera across the GIT regions belonging to bacteria, archaea, and eukaryotes. C, Candidatus. Only the dominant phyla or genera with a mean relative abundance greater than 1% at one site were listed. RUM, rumen; RET, reticulum; OMA, omasum; ABO, abomasum; DUO, duodenum; JEJ, jejunum; ILE, ileum; CEC, cecum; COL, colon; REC, rectum

To explore the segmental organization of microbial communities, we compared our gene catalog in 10 different regions and found that the GIT samples were partitioned into three distinct clusters, corresponding to different physiological areas, including the four-chambered stomach, small intestine, and large intestine (Fig. 1a). Notably, we found that the gastrointestinal microbiota exhibited a stable differential distribution along the GIT without modification by diet. Principal coordinate analysis (PCoA) revealed that region exerted a more pronounced effect on the separation of GIT microbial composition than diet (PERMANOVA, *F*_region_ = 27.2, *F*_diet_ = 9.4, *F*_region_ × *F*_diet_ interaction = 1.5, *p* < 0.05). This diet-independent pattern was observed in the alpha diversity index, which was highest in the four-chambered stomach (rumen, reticulum, omasum, and abomasum) and lowest in the small intestine (duodenum, jejunum, and ileum) (Wilcoxon rank-sum test, *p* < 0.01; Fig. 1b and Additional file 2: Fig. S1a). In addition, beta diversity was lowest in the four-chambered stomach, demonstrating an opposite trend to alpha diversity across the GIT regions in the forage-fed cows. However, no difference was observed between the four-chambered stomach and large intestine (cecum, colon, and rectum) of cows fed a grain-based diet (Fig. 1c and Additional file 2: Fig. S1b), which may be due to greater microbiome dissimilarity in the four-chambered stomach of grain-fed cows.

We next characterized the prevalent taxa of the GIT microbiome and similarly found region-specific patterns independent of diet regimes (Additional file 1: Table S2, S3). The GIT microbiome was mostly assigned to bacterial taxa, which were dominated by Firmicutes (46.5%), Bacteroidetes (33.6%), Proteobacteria (7.5%), and Fibrobacteres (3.0%) (Additional file 2: Fig. S2). The taxa classified into Bacteroidetes and Fibrobacteres were dominant in the four-chambered stomach, whereas the Firmicutes and Proteobacteria taxa were dominant in the post-gastric intestine (the small and large intestine) (Fig. 1d). *Prevotella* spp. and *Fibrobacter* spp. were enriched in the four-chambered stomach, *Phyllobacterium* spp. and *Eubacterium* spp. were more abundant in the small intestine, and *Bacteroides* spp. and *Alistipes* spp. were prevalent in the large intestine (Wilcoxon rank-sum test, *p* < 0.05) (Fig. 1f and Additional file 1: Table S3). Euryarchaeota occupied 99.5% of the annotated sequences that were assigned to archaea among the GIT regions (Wilcoxon rank-sum test, *p* < 0.05) (Fig. 1d and Additional file 1: Table S2). Notably, *Methanobrevibacter* spp. were the most prevalent archaea across all GIT regions and were mainly enriched in the post-gastric intestine, whereas *Methanocorpusculum* spp. were enriched in the large intestine (Fig. 1f). Nematoda were the dominant eukaryotic taxa in the GIT, with a relatively higher abundance in the post-gastric intestine (Fig. 1e). The top genera belonging to ciliates were *Oxytricha* spp., *Stylonychia* spp., and *Entodinium* spp., which were prevalent in the four-chambered stomach, whereas *Anaeromyces* spp. and *Caecomyces* spp., classified as Neocallimastigomycota, were relatively enriched in the large intestine (Fig. 1f). These results indicate that the GIT microbiome has a region-specific distribution across the physiological regions of dairy cattle, which represents segmental enrichment of distinct functional taxonomic groups, and may be related to polysaccharide utilization, H_2_ transfer, and fiber degradation in the GIT.

### Microbial functional landscape along the GIT of dairy cattle

To characterize the microbial function of the GIT microbiome in dairy cattle, we performed predictive analyses of metabolic enzymes, focusing on 7861 KOs and 335 CAZyme families from the GIT microbial gene catalog. We first investigated the core metabolic pathways, wherein KOs were present in at least 90% of the four-chambered stomach, small intestine, and large intestine samples. Of these, 377 core pathways from 1989 KOs were shared by the whole GIT microbiome, including biosynthesis of cofactors, ABC transporters, carbon metabolism, biosynthesis of amino acids, two-component systems, and methane metabolism (Additional file 1: Table S4). When exploring specific KOs in the four-chambered stomach (788), small intestine (78), and large intestine (193), we observed enrichment of purine metabolism, N-glycan biosynthesis, and glycerophospholipid metabolism in the four-chambered stomach; enrichment of oxidative phosphorylation, arachidonic acid metabolism, and tryptophan metabolism in the small intestine; and enrichment of antibiotic biosynthesis, galactose metabolism, carbon metabolism, and amino acid metabolism in the large intestine (Additional file 1: Table S4). These results suggest that the GIT microbiome exhibits substantial functional heterogeneity. Notably, we found segmental differences in the prokaryotic ABC transporters associated with transporting carbon-derived nutrients. For example, the four-chambered stomach microbiome seems to be particularly responsible for transporting cellobiose, L-arabinose, lactose, alpha-glucoside, sorbitol, mannitol, mannose, and glucose. Those in the large intestine were particularly associated with trehalose, maltose, and fructose transport, and no specific transporters were found in the small intestinal microbiome (Additional file 1: Table S4). These results highlight that the GIT microbiome of dairy cattle has a region-specific prevalence of trophic transport systems, which are associated with differences and complexities of nutrient substrates in distinct GIT regions.

**Fig. 2:**
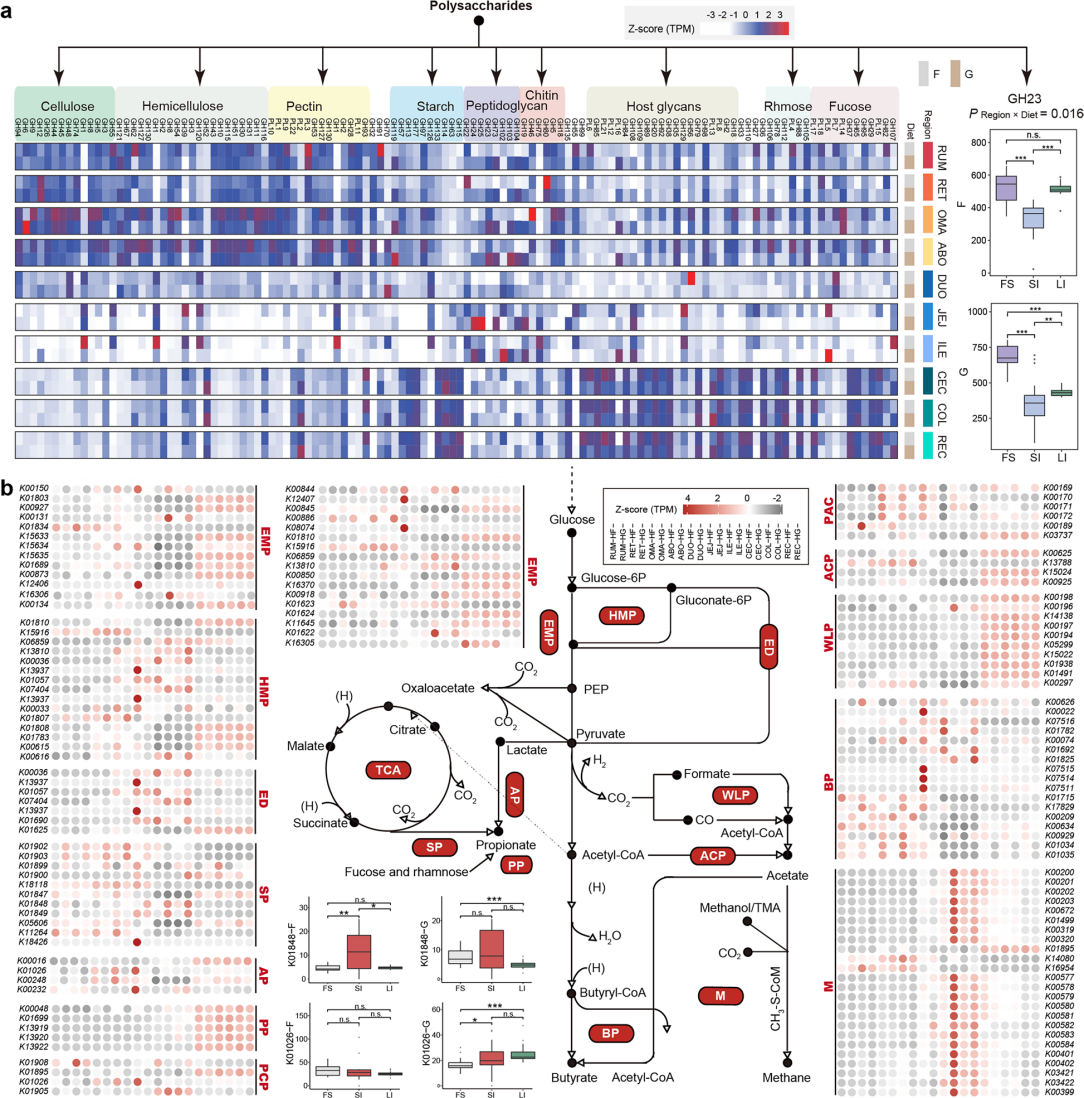
Spatial variation of polysaccharide degradation and fermentation pathways in the gastrointestinal tract (GIT) microbiome in forage-based (F) and grain-based (G) diets, respectively. **a** Comparing the abundance of glycoside hydrolase (GH) and polysaccharide lyase (PL) families assigned to the relevant substrates of the microbiome in the 10 GIT regions, and a *Z*-score was used to correct abundance. The region-specific distribution of the peptidoglycan-degrading gene GH23 between the F and G diets was compared (Scheirer–Ray–Hare test; *p*_Region × Diet_ = 0.016). **b** Comparison of KO levels in carbon metabolism modules of the microbiome in 10 GIT regions; a *Z*-score was used for correcting abundance. The comparison of the region-specific distributions of K01848 (*mcmA1*) and K01026 (*pct*) between the F and G diets (Scheirer–Ray–Hare test; *p*_Region × Diet_ < 0.05). EMP, embden-meyerhof-parnas; HMP, hexose monophosphate pathway; ED, Entner-Doudoroff pathway; SP, succinate pathway; AP, acrylate pathway; PP, propanediol pathway; PCP, propionyl-CoA to propionate; PAC, pyruvate to acetyl-CoA; ACP, acetyl-CoA to acetate; WLP, Wood–Ljungdahl pathway; BP, butyrate production; M, methanogenesis. RUM, rumen; RET, reticulum; OMA, omasum; ABO, abomasum; DUO, duodenum; JEJ, jejunum; ILE, ileum; CEC, cecum; COL, colon; REC, rectum

Ruminant GITs possess an efficient microbial polysaccharide degradation system in which many glycoside hydrolases (GHs) and polysaccharide lyases are involved [11]. Thus, we characterized region-specific features of polysaccharide degradation among the GIT regions. We first assigned 761,245 CAZymes to 119 GH (95.7%) and 22 polysaccharide lyase (4.3%) families and then classified them into functional groups based on their main polysaccharide targets (Fig. 2a and Additional file 1: Table S5). We found that enzymes belonging to the amylase family, GH13, and hemicellulase families, GH43 and GH3, were the three most prevalent across all GIT segments. Interestingly, we found that most CAZyme families had a region-specific distribution, regardless of the diet regime. For example, the cellulase, hemicellulase, and pectinase families showed a higher representation in the four-chambered stomach (Wilcoxon rank-sum test, *p* < 0.001). Microbe-derived glycans (e.g., peptidoglycan and chitin) were likely important substrates for the microbiota in the small intestine, which was supported by the enrichment of lysozyme (GH25 and GH24) and chitosanase (GH19). Notably, most host glycan-degrading families (e.g., GH109, GH92, and GH20) were enriched in the large intestine. However, the major peptidoglycan-degrading family (GH23) did not differ between the microbiomes of the four-chambered stomach and large intestine in forage-fed cows but was most prevalent in the four-chambered stomach under a grain-based diet (Scheirer–Ray–Hare test; *p*_Region × Diet_ = 0.016), which may be due to the grain introduction, leading to the accumulation of microbe-derived glycans in the four-chambered stomach (Fig. 2a and Additional file 1: Table S5). Together, these findings indicate the region-specific features of polysaccharide degradation in the GIT microbiome caused by the geographic specialization in nutrient availability and taxonomic populations of different regions [3, 6].

We hypothesized that the carbohydrate fermentation strategies of region-specific microbiomes also have an adaptive pattern. The fermentation stoichiometries of volatile fatty acids were first detected, and a higher proportion of acetate in the large intestine than in the four-chambered stomach and greater proportions of propionate and butyrate in the four-chambered stomach were observed (Additional file 2: Fig. S3). We further explored the features of carbohydrate fermentation in the GIT microbiome (Fig. 2b and Additional file 1: Table S6). A relatively high abundance of genes involved in converting acetyl-CoA to acetate and the Wood–Ljungdahl pathway was found in the large intestine. In addition, genes (*fucO* and *pduCDEP*) related to the propanediol pathway, one of the propionate biosynthesis pathways via deoxyhexose [12], were elevated in abundance in the large intestine, which was linked to the enrichment of fucose-degrading enzymes (e.g., GH29 and GH37) and rhamnose-degrading enzymes (e.g., GH78 and GH88) in the large intestine (Additional file 1: Table S5). We also observed higher representation of the gene encoding phosphate butyryltransferase (*ptb*), which catalyzes butyryl-CoA to butyrate in the four-chambered stomach. Interestingly, methanogenesis genes, including *fwdABCD*, *ftr*, *mch*, *mtd*, *mer*, *mtrABCDEFGH*, and *mcrABCDG*, were more abundant in the jejunum and ileum, further emphasizing the possibility of methanogenesis in the small intestine. Together, these findings revealed segmental differences in microbial functional groups based on the fermentation patterns in the GIT.

### Overview of 3079 draft microbial genomes constructed from dairy cattle GITs

**Fig. 3:**
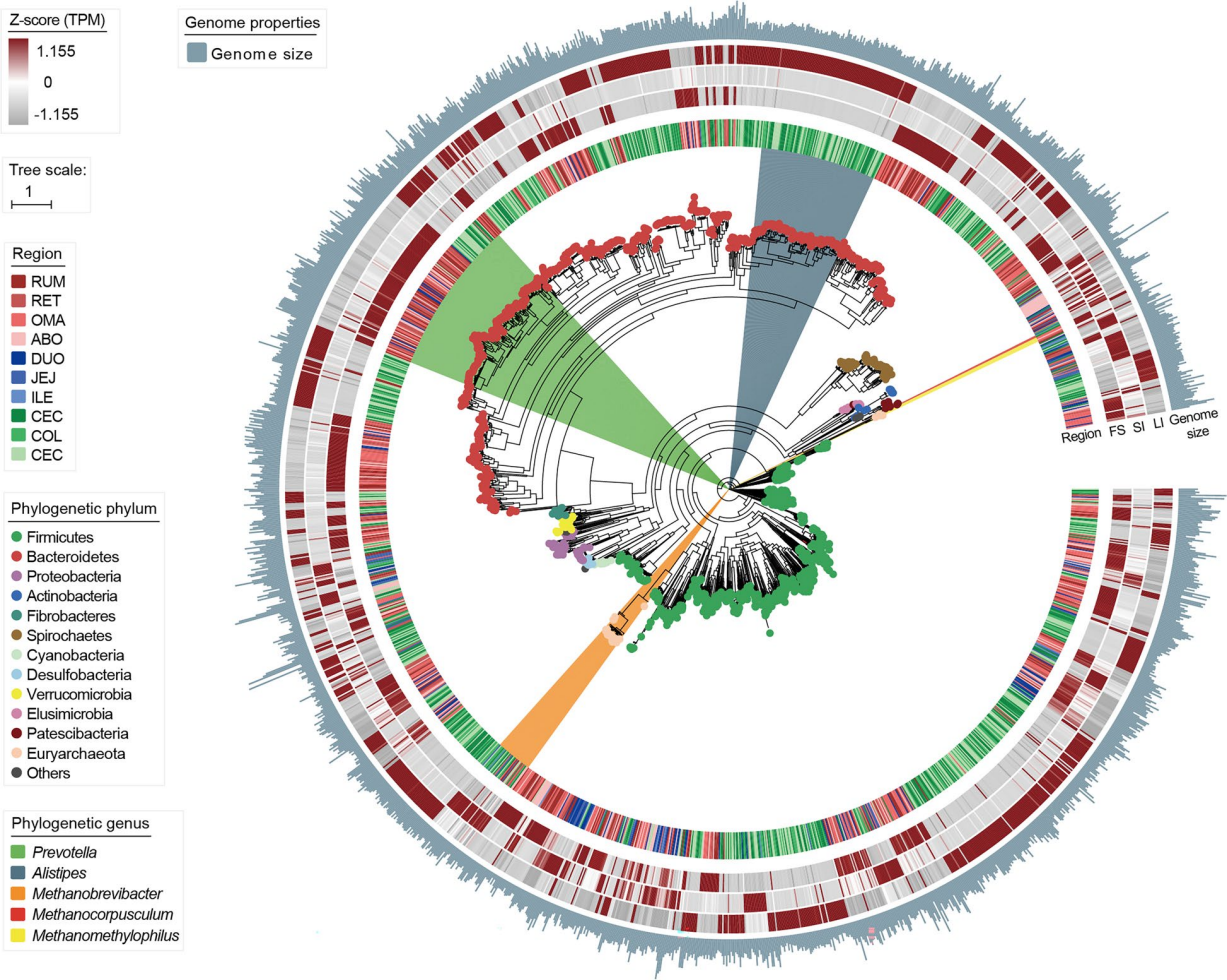
Phylogenetic tree of 1904 strain-level genome bins (SGBs) sampled from the gastrointestinal tract (GIT) regions of dairy cattle. The maximum-likelihood tree was constructed using PhyloPhlAn. Dots on the clades of the tree are colored according to phyla. Branches are shaded with color to highlight genus-level affiliations. The inside layers of the heat map represent the SGBs from different GIT regions. The outside layers of the heat map represent the abundance of each genome in the FS, SI, and LI groups, and a *Z*-score was used for correction. FS, four-chambered stomach; SI, small intestine; LI, large intestine; RUM, rumen; RET, reticulum; OMA, omasum; ABO, abomasum; DUO, duodenum; JEJ, jejunum; ILE, ileum; CEC, cecum; COL, colon; REC, rectum

To further clarify microbial organization and functionality at the genomic level, we performed contig binning based on single-sample assemblies and obtained 23,356 MAGs. After quality control and data filtration, 3079 MAGs exceeding medium quality (completeness ≥ 50% and contamination ≤ 10%) remained, of which 1450 were from the four-chambered stomach, 249 were from the small intestine, and 1380 were from the large intestine (Additional file 1: Table S7). We then compiled 1904 strain-level genome bins (SGBs) with a dereplication cutoff of 99% average nucleotide identity (Additional file 1: Table S8). Of these, 592 SGBs were of high quality (completeness > 80%, contamination < 10%, and quality score > 50). For taxonomic profiling, 323, 1507, and 1904 SGBs were classified into microbes at the species, genus, and phylum levels, respectively (Additional file 2: Fig. S4a). PCoA analysis showed that MAG profiling presented clear divergence among the four-chambered stomach, small intestine, and large intestine, as described in the gene catalog data (Additional file 2: Fig. S4b). At the phylum level, 15 phyla were annotated, mainly consisting of Firmicutes (861) and Bacteroidetes (747), followed by Spirochaetes (76), Proteobacteria (61), and Euryarchaeota (39) (Additional file 2: Fig. S4a). Among them, 1865 SGBs mainly belonged to bacteria (47.4% of MAGs from the four-chambered stomach, 8.1% from the small intestine, and 44.5% from the large intestine) and 39 SGBs (28.3% of MAGs from the four-chambered stomach, 8.7% from the small intestine, and 63.0% from the large intestine) were classified as members of archaea (Fig. 3). Of the bacterial SGBs, most of the genomes were classified into the genera *Prevotella* (142 SGBs; 74.9% of MAGs from the four-chambered stomach) and *Alistipes* (106 SGBs; all MAGs from the large intestine). Furthermore, most SGBs belonging to the class Negativicutes consisting of propionate-producing bacteria via the succinate pathway [13] were enriched in the four-chambered stomach. In contrast, all 16 SGBs assigned to Kiritimatiellae were over-represented in the small intestine (Additional file 1: Table S9). Notably, 33 *Methanobrevibacterr*-affiliated SGBs, hydrogenotrophic methanogens [14], were mainly retrieved from the large intestine (60.6%). However, their abundance was higher in the small intestine (Fig. 3). We also observed the prevalence of four *Methanomethylophilus*-affiliated SGBs (methylotrophic methanogens) in the four-chambered stomach and the representation of two *Methanocorpusculum*-affiliated SGBs (hydrogenotrophic methanogens) in the large intestine [14, 15] (Additional file 1: Table S9). These results suggest that different taxonomic groups have region-specific distributions across the GIT, and these inferences may be confirmed by genomic function analysis.

### Genome-based characterization of glycan-degrading microbes across GIT regions

We further compared the CAZyme profiles of 592 high-quality SGBs from different GIT regions. The cellulosomal components were first investigated, and we found that dockerin-containing proteins were widely identified in five phyla, dominated by Firmicutes (51.7%) and Bacteroidetes (46.3%), and 36.9% of them possessed cohesion modules (Additional file 1: Table S10). By comparing the genomic abundance among the GIT microbiomes, we found that most four-chambered stomach-enriched SGBs were classified as *Prevotella* spp. (e.g., *Prevotella* sp. UBA3846) and *Ruminococcus* spp., which may have the potential to produce cellulosomes because of the prevalence of dockerin and cohesion, as well as cellulase (e.g., GH5 and GH9), and hemicellulose (e.g., GH43) (Additional file 1: Table S10), indicating that the microbes in the four-chambered stomach have a powerful potential to degrade fiber [7, 16, 17]. In addition, we found that the four-chambered stomach-enriched Muribaculaceae C941 spp. had a prevalence of dockerin and GH13 but without cohesion (Additional file 1: Table S10). Members of Muribaculaceae C941 likely contribute to starch degradation via a non-amylosomal approach in the four-chambered stomach. In contrast, the hindgut-enriched SGBs belonging to *Alistipes* spp. lacked dockerin modules, and the Kiritimatiellae-affiliated SGBs that were enriched in the small intestine encoded myriad GH109 enzymes to degrade glycans derived from host mucins [18] (Additional file 1: Table S10). These results indicate that microbes placed in the four-chambered stomach provide efficient hydrolysis for complex glycan breakdown to deal with primary dietary substrates.

**Fig. 4:**
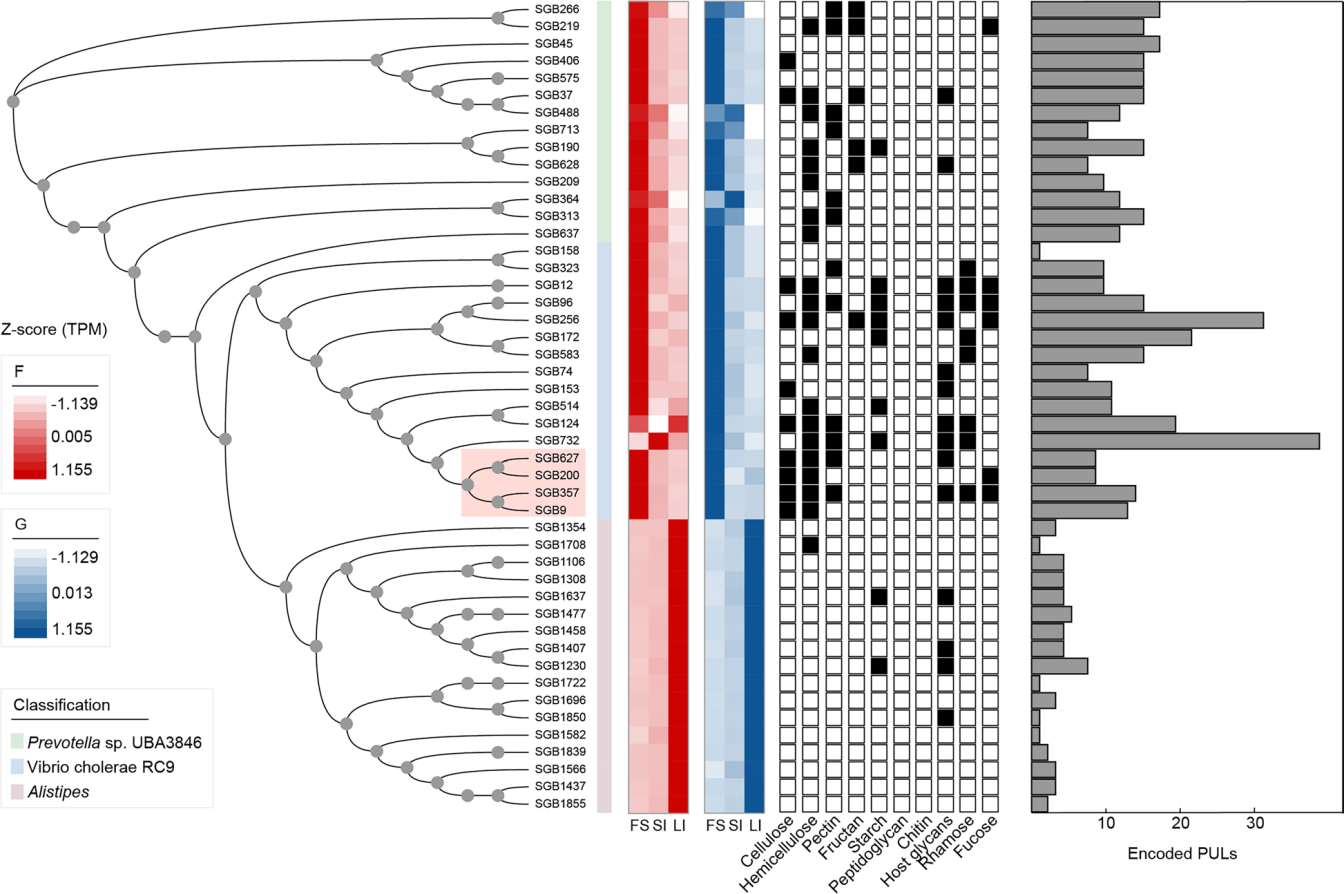
Detection and trait of polysaccharide utilization loci (PULs) across *Prevotella* sp. UBA3846, Vibrio cholerae RC9, and *Alistipes* members. Maximum-likelihood phylogenetic tree of 47 high-quality strain-level genome bins (SGBs) recovered. Lines connecting the tree to the genome name are colored by taxonomic family assignment. Circles represent nodes with bootstrap support greater than 70 (out of 100 bootstraps). Heatmaps showing a comparison of genomes in abundance across the four-chambered stomach (FS), small intestine (SI), and large intestine (LI), and a *Z*-score was used for correcting abundance. The black boxes indicate that at least one gene within the specific substrate-degrading PUL was detected. Histograms colored in gray indicated the number of PULs encoded from genomes. F, forage-based diet; G, grain-based diet

We further predicted the polysaccharide utilization loci (PULs) for these SGBs and found that the four-chambered stomach-enriched *Prevotella* sp. UBA3846 also possessed more than 12 PULs (Additional file 1: Table S11) that were predicted to encode various polysaccharide-degrading enzymes, such as hemicellulase (e.g., GH43 and GH3), fructan-degradation (e.g., GH32), and pectinase (e.g., GH28) PULs (Fig. 4). These results highlight that the enrichment of *Prevotella* spp. in the four-chambered stomach may serve as a strong force for degrading complex glycans. Other four-chambered stomach-enriched SGBs (SGB9, SGB200, SGB357, and SGB627) belong to Vibrio cholerae RC9 encoded PULs that contained double cellulase GH26 (Fig. 4 and Additional file 2: Fig. S5), indicating its potential for degrading low-value plants in the four-chambered stomach. In contrast, the hindgut-enriched *Alistipes*-affiliated SGBs (SGB1850, SGB1637, SGB1230, and SGB1407) utilized host-derived glycans inferred by the enrichment of GH109, GH20, and GH92 genes and encoded putative PULs that also contained GH20, GH2, and GH27 CAZymes (Fig. 4 and Additional file 1: Table S10). These findings support the idea that many host mucous secretions and shed epithelial cells serve as additional energy sources for microbes in the hindgut of dairy cattle. Together, the four-chambered stomach microbiome orients toward the degradation of plant-derived glycans and the microbiome in the post-gastric intestine can utilize host-derived glycans.

### Genome-based characterization of hydrogen metabolism–associated microbes across GIT regions

**Fig. 5:**
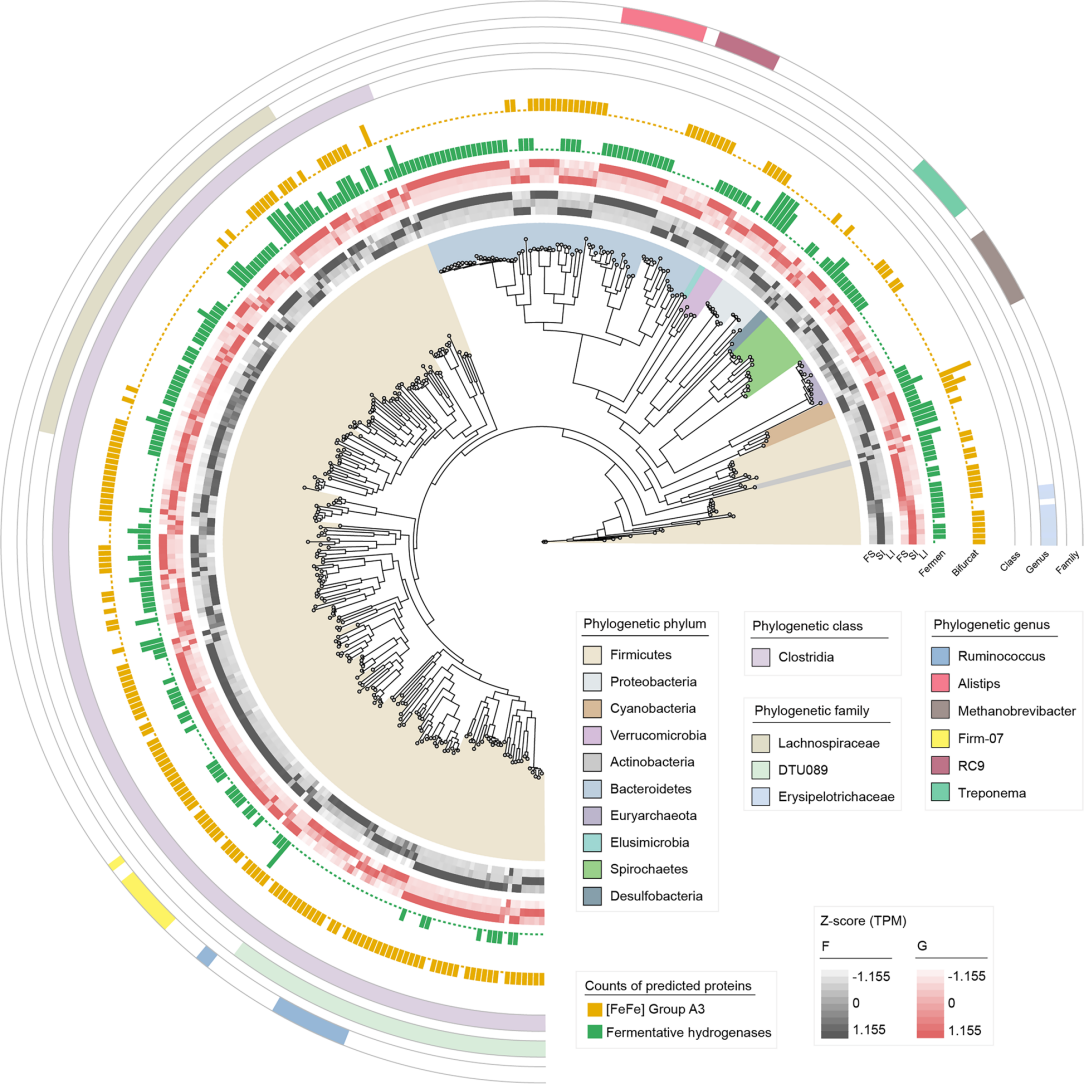
Phylogenetic tree of 358 high-quality strain-level genome bins (SGBs) encoding the capacity to metabolize H_2_ via [FeFe]-hydrogenases and [NiFe]-hydrogenases sampled from the gastrointestinal tract (GIT) regions of dairy cattle. Fermen, fermentative hydrogenases (group A1, A2, and B [FeFe]-hydrogenases); Bifurcat, electron-bifurcating hydrogenases (group A3 [FeFe]-hydrogenases). F, forage-based diet; G, grain-based diet

Hydrogen can be primarily produced via microbial fermentation processes by hydrogenases, which are also the major substances in hydrogenotrophic consortia, including methanogens, acetogens, fumarate reducers, sulfate reducers, and nitrate reducers [2]. Here, we focused on microbial populations possessing hydrogenases ([NiFe]-, [FeFe]-, and [Fe]-hydrogenases) among 592 high-quality SGBs. We found that 358 SGBs classified into 10 phyla encoded [FeFe]- and [NiFe]-hydrogenases (Fig. 5), suggesting that H_2_ metabolism is a widespread trait among the GIT microbiome of dairy cattle. We observed that more hydrogenase-encoding genomes were obtained from the large intestine (53.1%), followed by the four-chambered stomach (37.7%) and small intestine (9.2%). Of these 358 SGBs, 213 encoded groups A1, A2, and B [FeFe]-hydrogenases for H_2_ evolution during fermentation (Fig. 5 and Additional file 1: Table S12), and approximately 59.2% of the MAGs were obtained from the large intestine. Further considering the microbiome of the large intestine, we found that these fermentative hydrogenases in the large intestine were uniquely contributed by *Alistipe* spp. (Fig. 5). Interestingly, up to 54.5% of hydrogenases were group A3 [FeFe]-hydrogenase (Fig. 5 and Additional file 1: Table S13), suggesting that such electron-bifurcating hydrogenases appear to substantially mediate H_2_ production by the GIT microbiome in dairy cattle [2]. Over half of the MAGs (55.9%) within the coding group A3 [FeFe]-hydrogenase were obtained from the large intestine. The majority of electron-bifurcating hydrogenases were contributed by the genera *Ruminococcus*, *Vibrio cholerae RC9*, and *Clostridiales bacterium Firm_07* (Fig. 5). The contributors also showed segmental heterogeneity, such as the main contribution of the facultative anaerobic fermentative bacteria Erysipelotrichaceae in the small intestine (Fig. 5). Therefore, the analysis of [FeFe]-hydrogenases indicates that H_2_ production in the GIT microbiome is primarily driven by fermentative and electron-bifurcating hydrogenases and that microbial populations in the large intestine may contribute to greater H_2_ evolution.

We further identified 11 methanogen SGBs that processed hydrogenotrophic methanogenesis by encoding H_2_-uptake [NiFe]-hydrogenases (groups 3a, 3c, 4 h, and 4i) and methyl-CoM reductases (*mcrA*), including *Methanobrevibacter* spp. (20), *Methanocorpusculum* spp. (2), and *Methanomethylophilus* (1) (Additional file 3: Table S14). Methanogenic hydrogenases were mainly contributed by the genomes of the large intestine (54.5%). We also noticed other hydrogen-utilizing functional groups that harbored the required terminal reductases and specific hydrogenases to compete for H_2_ with hydrogenotrophic methanogens. Two SGBs (SGB1248 and SGB1432) from the large intestine (Lachnospiraceae) encoded both [FeFe]-hydrogenases (A3 or A4) and the marker genes for hydrogenotrophic acetogenesis (*acsB*, *CooS*, *cdhD*, *cdhE*, or *FdhA*). Fumarate reduction was driven by *Acetobacter* spp., *Selenomonas* spp., and *Escherichia* spp. within the coding [NiFe]-hydrogenase (groups 1d or 1c) and fumarate reductase (*SdhA*), and 66.7% of the contributors were obtained from the four-chambered stomach. *Desulfovibrio*-affiliated SGB901 encoded group 1b [NiFe]-hydrogenase and terminal reductases *AprA*, *DsrA*, and *NrfA* for sulfate and nitrate reduction. Together, distinct GIT habitats support different hydrogen-utilizing functional groups.

### Region-specific responses of microbial populations to a grain-based diet introduction

**Fig. 6:**
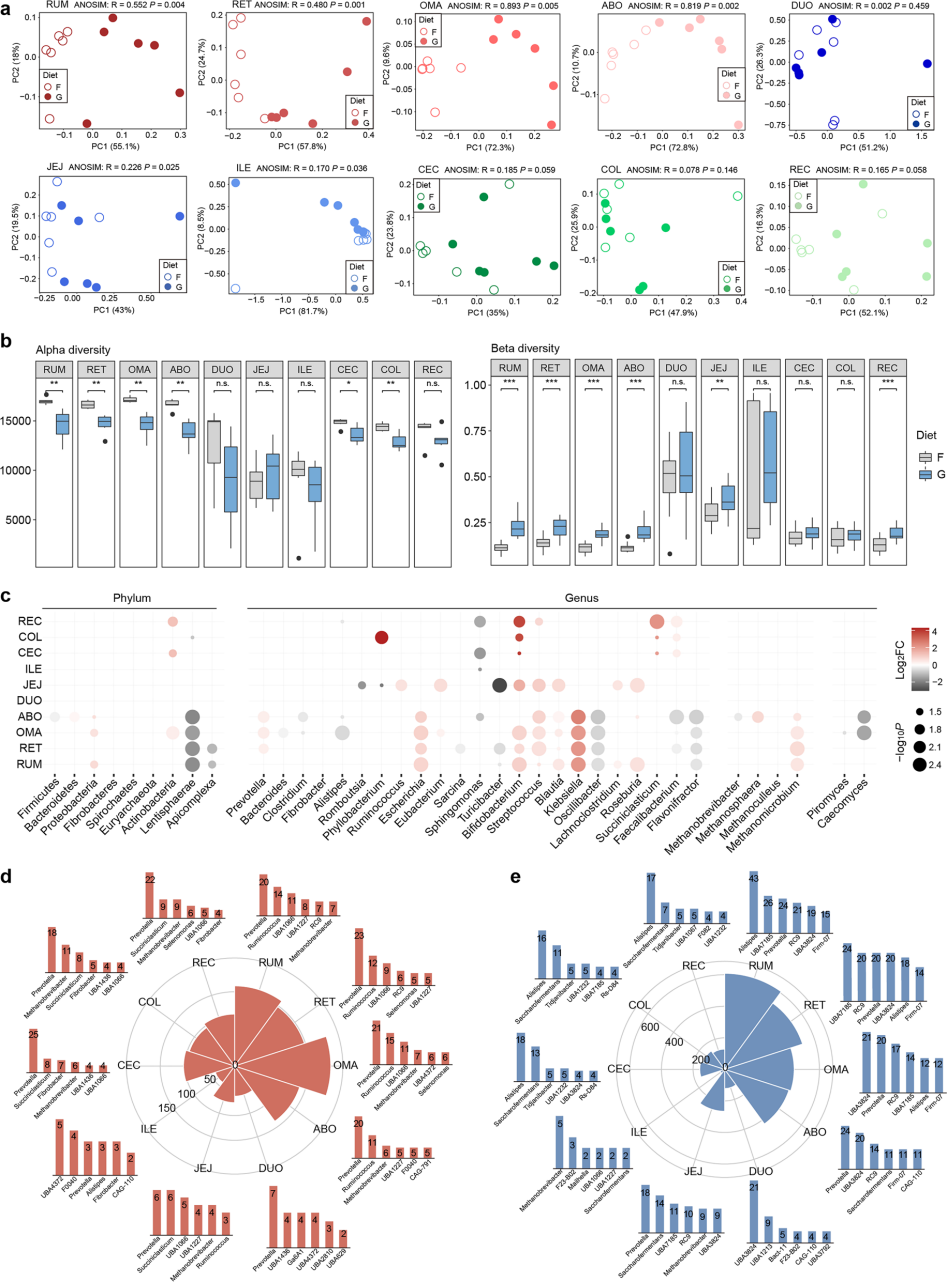
**a** Principal coordinate analysis (PCoA) plot of the gene catalog at the genus level between the forage-based (F) and grain-based (G) diets in each gastrointestinal tract (GIT) region. The differences between groups were assessed using the ANOSIM test based on the Bray–Curtis dissimilarity index. **b** Comparison of alpha diversity (richness index) and beta diversity (Bray–Curtis) between the F- and G-fed animals, respectively. Significance is based on the relative index of each cohort according to the Wilcoxon rank-sum test. *p < 0.05, **p < 0.01, ***p < 0.001. **c** The different abundances in dominant taxa at the phylum and genus levels of the bacterial, archaeal, and eukaryotic communities among the GIT regions between the F and G diets. The number and top taxonomic populations of the significantly increased (**d**) and decreased (**e**) abundances of SGBs from the proximal to distal GIT (Wilcoxon rank-sum test, log_2_^fold−change^ > 1 and *p* < 0.05). The height of the column represents the number of differential SGBs classified into specific genera. RUM, rumen; RET, reticulum; OMA, omasum; ABO, abomasum; DUO, duodenum; JEJ, jejunum; ILE, ileum; CEC, cecum; COL, colon; REC, rectum

To further illustrate the region-specific responses of the GIT microbiota to a diet regime shift in dairy cattle, we compared the changes in microbial taxa at both the gene and genome levels between forage- (control) and grain-based diets. Although dietary regimes had little influence on the segmental dissimilarity of the GIT microbiome, we found that grain introduction significantly affected the GIT microbial structure in each region, particularly for the four-chambered stomach microbiome, which preferentially consumed dietary substrates (ANOSIM, *p* < 0.01; Fig. 6a). The decreased richness and increased inter-individual variability among the four-chambered stomach and large intestine (Fig. 6b) suggest that a grain-based diet drives the instability of a microbial community, resulting in the loss of specific taxa and greater microbiome dissimilarity between individual animals.

We found that a grain-based diet markedly affected taxonomic populations in the four-chambered stomach with the expansion of Proteobacteria and the reduction of Lentisphaerae (Fig. 6c; Additional file 1: Table S15). A significantly increased abundance of the dominant taxa *Prevotella* was observed in the four-chambered stomach of grain-fed cows. Elevation of *Methanomicrobium* spp. in the four-chambered stomach and reduction of *Methanobrevibacter* spp. in the abomasum occurred in grain-fed cows. A grain-based diet also reduced the relative abundance of *Piromyces* spp. and *Caecomyces* spp. classified as fiber-degrading fungi Neocallimastigomycota in the omasum. The jejunum was the most affected region in the small intestine, seeing effects such as evidenced by enrichment of the genus *Ruminococcus* and depletion of the genera *Romboutsia*, *Phyllobacterium*, and *Turicibacter*. The genera *Faecalibacterium*, *Bifidobacterium*, and *Succiniclasticum* were significantly elevated in the hindgut, whereas the dominant genus *Alistipes* was reduced in the rectum.

At the genome level, we observed that the genomic abundance of *Prevotella* spp. shifted across the GIT regions after grain introduction (Wilcoxon rank-sum test, log_2_^fold−change^ > 1 and *p* < 0.05; Additional file 1: Table S16). In the four-chambered stomach, we found that a grain-based diet significantly increased the abundance of *Prevotella* and *Ruminococcus*-affiliated SGBs (Fig. 6d), whereas it generally decreased the abundance of SGBs belonging to *Alistipes* spp. and Peptococcaceae bacterium UBA7185 (Fig. 6e). In the post-gastric GIT, the abundance of *Prevotella*-affiliated SGBs increased (Fig. 6d), whereas the abundance of *Alistipes* and *Saccharofermentans*-affiliated SGBs decreased in the large intestine (Fig. 6e). These results indicated that a grain-based diet selectively alters diverse microbial populations in different GIT regions.

### Region-specific modification of metabolic cascades of the GIT microbiome by a grain-based diet

We further outlined that segmental variations of major metabolic cascades from macromolecules undergo polysaccharide degradation and fermentation in which metabolites are transferred among microbes. We found that the microbiota in the four-chambered stomach and small intestine was oriented toward propionate-type fermentation (*t*-test, *p* < 0.05), whereas no change in fermentation type occurred in the hindgut (Additional file 2: Fig. S6). We hypothesized that the carbon-fueled trophic structure would change under a grain-based diet, including polysaccharide degradation and glucose fermentation.

To test this hypothesis, we explored the microbial potential for polysaccharide degradation after a diet regime shift. A grain-based diet caused a mass of changes in the abundance of CAZyme families across the GIT regions, most of which were associated with the degradation of starch, plant cell wall, and microbial cell wall (Additional file 1: Table S17). In the four-chambered stomach, a grain-based diet increased the abundance of peptidoglycan-degrading families GH73, GH103, GH104, GH23, GH24, GH25, alpha-amylase family GH119, and chitinase family GH19 (Additional file 1: Table S17). Higher peptidoglycan-degrading ability was also observed in the small intestine (GH104) and large intestine (GH24 and GH25) (Additional file 1: Table S18). In contrast, a grain-based diet reduced the abundance of the cellulose-binding enzyme CBM9 in both the four-chambered stomach and large intestine (Additional file 1: Table S19). Together, a grain-based diet greatly changed the degradation strategies from polysaccharide to glucose by the microbial enzymatic repertoire in the GIT of dairy cattle.

**Fig. 7:**
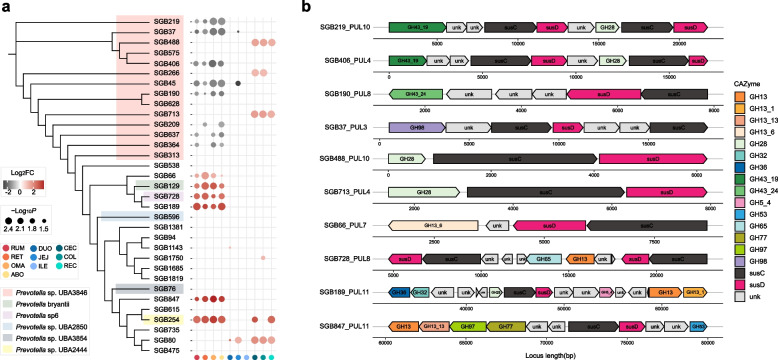
Phylogenetic tree of *Prevotella*-affiliated genomes and its association with polysaccharide utilization loci (PULs) in the distinct diets. **a** Maximum-likelihood tree of the 33 high-quality *Prevotella*-affiliated genomes constructed using PhyloPhlAn. The bubble diagram indicates relative changes in the abundance of *Prevotella* genomes within two diets across the whole gastrointestinal tract (GIT). **b** Schematic representation of predicted PULs in targeted *Prevotella* genomes. RUM, rumen; RET, reticulum; OMA, omasum; ABO, abomasum; DUO, duodenum; JEJ, jejunum; ILE, ileum; CEC, cecum; COL, colon; REC, rectum

To further dissect the assignment of specific taxa to the polysaccharide degradation system after grain introduction, we focused on the microbial populations within the broader substrate-related enzymatic repertoire. Strikingly, a grain-based diet significantly depleted the abundance of SGBs affiliated with cellulose-degrading members of *Prevotella* sp. UBA3846 in the four-chambered stomach (Fig. 7a and Additional file 1: Table S16), implying a reduction in fiber degradation ability in the four-chambered stomach. A closer examination of *Prevotella* sp. UBA3846 revealed that the reduction of several genomes (SGB219, SGB190, and SGB406) in the four-chambered stomach possessed GH43-containing hemicellulase and GH28-containing pectinase PULs, whereas SGB488 and SGB713 were found to encode GH28-containing pectinase PUL and were elevated in the large intestine (Fig. 7b). In addition, the expansion of other *Prevotella* spp. (e.g., SGB847, SGB189, SGB728, and SGB66) in the four-chambered stomach was predicted to possess diverse amylase PULs (e.g., GH13-GH13_13-GH97-GH77 and GH13-GH13_1) (Fig. 7b). Therefore, a grain-based diet changes the microbial environment in the four-chambered stomach, which regulates taxonomic reassembly in favor of starch degradation by narrowing down plant biomass hydrolysis, and excessive pectin is mainly degraded in the large intestine.

Regarding glucose fermentation pathways, we found that a mass change in KOs occurred in the four-chambered stomach after feeding a grain-based diet (Additional file 2: Fig. S7). For the glycolytic pathways, the expansions of polyphosphate glucokinase (*ppgK*) involved in the embden-meyerhof-parnas pathway, and ribose 5-phosphate isomerase A (*rpiA*) and ribulose-phosphate 3-epimerase (*rpe*) involved in the hexose monophosphate pathway were observed in the four-chambered stomach after grain-based diet feeding (Additional file 1: Table S20). Notably, the expansion of genes involved in the tricarboxylic acid cycle was observed in the four-chambered stomach, which suggests plentiful production of dicarboxylic acids after grain introduction [19] (Additional file 1: Table S20). The decarboxylation of dicarboxylic acids serves as the sole energy source for the growth of fermenting bacteria [20]; therefore, the expansion of the tricarboxylic acid cycle may support the acceleration of the fermentation process by a grain-based diet.

**Fig. 8:**
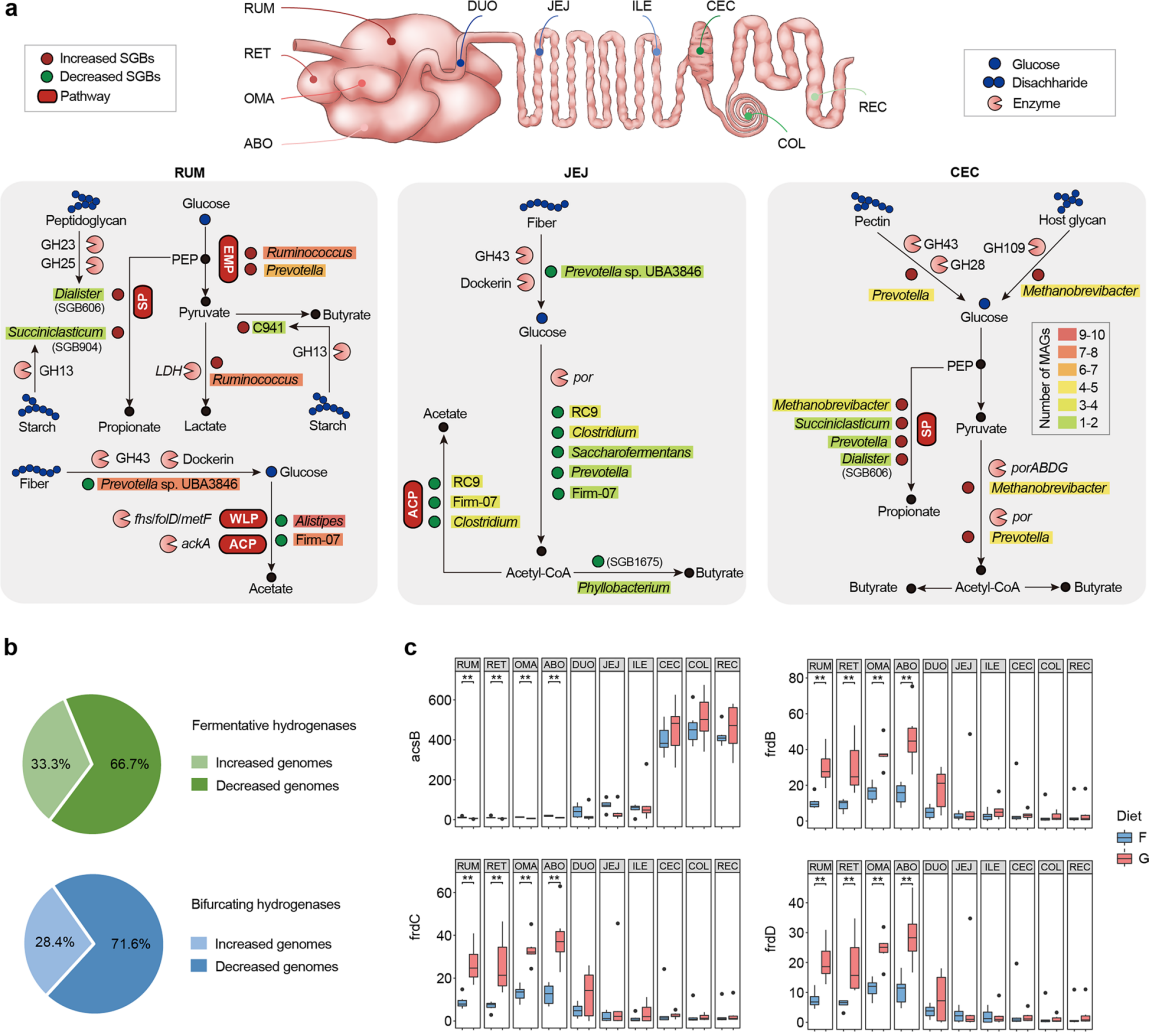
Changes in fermentation schemes of the gastrointestinal tract (GIT) microbiome during a grain-based diet challenge. **a** The different metabolic models (polysaccharide degradation and fermentation pathways) are displayed for significantly shifted strain-level genome bins (SGBs) in the rumen, jejunum, and cecum. Graphical representation of CAZymes, enzymes, genera, and pathways are based on functional annotations. The background colors of genera are based on the number of SGBs within significance. **b** The significantly shifted abundance of genomes encoded fermentative hydrogenases (group A1, A2, and B [FeFe]-hydrogenases) or electron-bifurcating hydrogenases (group A3 [FeFe]-hydrogenases) in at least one region in the grain-based diet. **c** Comparison of the specific terminal reductases and hydrogenotrophic acetogenesis (*acsB*) and fumarate reduction (*frdBCD*) between the forage-based (F) and grain-based (G) diets. Significance is based on the relative index of each cohort according to the Wilcoxon rank-sum test. *p < 0.05, **p < 0.01, ***p < 0.001. RUM, rumen; RET, reticulum; OMA, omasum; ABO, abomasum; DUO, duodenum; JEJ, jejunum; ILE, ileum; CEC, cecum; COL, colon; REC, rectum

Next, we assessed how microbial populations modified physiological fermentation schemes via representative segments (rumen, jejunum, and cecum) after grain-based diet feeding at the genome level (Fig. 8a). In the rumen, *Dialister*-affiliated SGB606 exhibited a 139-fold increase in abundance under the grain-based diet and possessed a complete succinate pathway (Additional file 4: Table S21), suggesting that it contributes largely to the higher propionate concentration after grain-based diet feeding (Additional file 2: Fig. S6). The substantial reduction in the abundance of members of *Alistipes* and Clostridiales bacterium Firm_07 also exhibited a high prevalence of genes controlling the conversion of acetyl-CoA to acetate (*ackA*) and genes involved in the Wood–Ljungdahl pathway (*fhs*, *fold*, and *metF*; Additional file 5: Table S22), thereby possibly creating significant effects on the reduction of acetate proportion (Additional file 2: Fig. S6). In the jejunum, we found that a decreased abundance of *por*-carrying SGBs (e.g., Vibrio cholerae RC9 and *Clostridium*) encoded the process of pyruvate conversion to acetyl-CoA (Additional file 6: Table S23). Thus, the suppressed microbial populations responsible for reducing pyruvate to acetyl-CoA were the main contributors to the decreased concentrations of acetate and butyrate in the jejunum (Additional file 2: Fig. S6). The cecum substantially increased the stoichiometry of volatile fatty acids, including the concentrations of acetate, propionate, and butyrate, which was different from that in the rumen and jejunum in that there were no changes in the ratio of acetate to propionate (Additional file 2: Fig. S6). Among the more abundant genomes, *Methanobrevibacter*-affiliated genomes encoded *porABDG* and *Prevotella*-affiliated genomes encoded *por* to promote the production of acetate and butyrate (Additional file 7: Table S24). Overall, grain-based diets influence region-specific microbial populations to reorganize microbial fermentation strategies in the GIT of dairy cattle.

### Reshuffle of hydrogenogenic and hydrogenotrophic processes during a grain-based diet

Hydrogen metabolism is a key junction that connects different microbial functional groups in the GIT ecosystem [13]. Hence, we further decoded the segmental heterogeneity of interspecies hydrogen transfer during shifts in the fermentation type after a grain-based diet. We observed that 66.7% of diet-altered genomes coding fermentative hydrogenases and 71.6% coding electron-bifurcating hydrogenases were decreased in abundance in grain-fed cows and were mainly classified into members of Clostridiales bacterium Firm_07, *Ruminococcus*, and *Alistipes* (Additional file 8: Table S25). In addition, the genomes encoding hydrogenases were mainly reduced in the four-chambered stomach, followed by those in the jejunum and large intestine. These results suggest that a grain-based diet may cause fewer H_2_ sinks across GIT regions [3, 21]. We speculated that a large proportion of genomes encoding H_2_-evolving hydrogenases that decreased after grain diet feeding may affect hydrogenotrophic pathways. To test this, we focused on the change in the abundance of genomes coding H_2_-uptake modules, including methanogenic hydrogenases (groups 3a, 3c, 4 h, and 4i [NiFe]-hydrogenases) and respiratory hydrogenases (groups 1d, 1c, and 1b [NiFe]- hydrogenases), accompanied by the required terminal reductases. As expected, the marker gene for hydrogenotrophic acetogenesis (acetyl-CoA synthase, *acsB*) was reduced in the four-chambered stomach after grain introduction (Fig. 8c and Additional file 1: Table S20). This result also underpinned the reduction of acetate-type fermentation in the four-chambered stomach and small intestine (Additional file 2: Fig. S6). Moreover, no changes were observed in the abundance of *mcrA* in the methanogenic pathway (Additional file 1: Table S20). Notably, we observed that a grain-based diet promoted the gene abundance of *frdBCD*, which was involved in fumarate reduction in the four-chambered stomach that underpinned the process of propionate production (Fig. 8c and Additional file 1: Table S20). Therefore, a grain-based diet may cause a more significant H_2_ sink in the propionate pathway than in other fermentation pathways.

## Discussion

The GIT microbiome of dairy cattle is a complex system in which microbial populations are region-specific with taxonomic and functional heterogeneity. Herein, we demonstrated that the alpha diversity of the GIT microbiome generally decreased from the four-chambered stomach to the small intestine and then increased in the large intestine. Interestingly, the opposite trend was observed for beta diversity from the proximal to the distal GIT. That said, the greater microbial richness and lower microbiome dissimilarity between individual animals could be explained by portfolio effects [13] that the increased biodiversity of the ecosystem creates a functional buffer against changing environmental conditions.

We next observed that the region-specific patterns of prevalent taxa in the entire GIT were independent of diet regime, which was further underpinned by the fact that differences in the GIT regions better explained the variance detected (63.6%) than diet did (1.3%). Of the taxonomic populations, the prevalence of *Prevotella* spp. and *Fibrobacter* spp. in the four-chambered stomach provides efficient hydrolysis for plant biomass conversion [22, 23], whereas the mucin-degrading taxa *Bacteroides* spp. [11] and *Alistipes* spp. were found in the large intestine. Additionally, we noticed that the dominant taxa of Firmicutes in the post-gastric intestine were *Clostridium* spp., whose members are commonly endospore-forming bacteria [24]. In addition, the spore-forming bacteria *Romboutsia* spp. and *Sarcina* spp. were enriched in the jejunum and ileum (Additional file 1: Table S3), and ciliates were almost nonexistent in the small intestine. As spore-forming bacteria have strong resistance and tolerance to environmental stress or restricted nutrients [25], these results suggest that escaping from low pH and host secretions by spore formation is an effective approach for microbial development in the small intestine. Furthermore, the aerobic bacteria *Phyllobacterium* spp., *Achromobacter* spp., and *Sphingomonas* spp. belonging to Proteobacteria were also relatively enriched in the small intestine (Additional file 1: Table S3). This observation matches the higher oxygen levels created by delivery from host tissues and oxygenation via pancreatic and biliary secretions [5]. In addition, members of Neocallimastigomycota were relatively enriched in the large intestine, suggesting the importance of Neocallimastigomycota with plant cell-wall decomposition in the large intestine of dairy cattle [26]. In addition, it is important to note that some microbes collected in different GIT regions may not be active inhabitants but simply passers, especially relevant for the abomasum and duodenum with fast passage rates and harsh environments. Altogether, biogeography is a deterministic filter used to select microbial populations via many factors, including nutrient availability, chemical gradients, oxygen tension, and host secretions [5, 27].

The region-specific microbial composition further results in the spatial distribution of their functions. Through annotated genes, we found that the prevalence of ABC transporters and carbon metabolism was shared among the GIT microbiome, accompanied by region-specific ABC transporters associated with the transport of carbon-derived nutrients. Furthermore, the enrichment of plant glycan-degrading genes in the four-chambered stomach, microbe glycan-degrading genes in the small intestine, and host glycan-degrading genes in the large intestine were extraordinary traits of carbon source availability across the GIT regions. Subsequently, the large intestine preferentially selected the Wood–Ljungdahl pathway for acetate production wherein a set of biochemical reactions are performed by acetogens [28], suggesting that the hindgut microbiome orchestrates alternative ways for carbon fixation and energy conservation. Together with the representation of the propanediol pathway in the large intestine, our results highlight that oligotrophic taxa in the hindgut of dairy cattle have characteristic fermentation routes to adapt to nutrient availability and energy acquisition. In contrast, the prevalence of methanogenesis was a distinct signature in the jejunum and ileum, underpinned by the prevalence of the genus *Methanobrevibacter* [3], indicating that the small intestinal microbiota may also contribute to methane release. Together, these findings reveal that the microbiome in the post-gastric intestine contributes to the feed efficiency of dairy cattle and environmental climate change.

The predicted cellulosomes and PULs further decrypted the underlying mechanism of the region-specific glycan degradation strategies employed by different taxa. The four-chambered stomach-enriched consortia encoded a larger number of dockerin-associated CAZyme genes, including *Ruminococcus* spp. and *Prevotella* spp. (e.g., *Prevotella* sp. UBA3846), although we did not test transcription levels in the present study. In contrast, the gut-enriched genomes (e.g., class Kiritimatiellae and genus *Alistipes*) orchestrated myriad CAZymes for host glycan degradation, yet lacked dockerin modules. In anaerobic environments, cellulosomes are tailored to multicomponent catalytic machines for recalcitrant polysaccharide substrates [16]. In addition, the high prevalence of cellulose-, hemicellulose-, pectin-, and fructan-degrading PULs encoded by the four-chambered stomach-enriched consortia and host glycan-degrading PULs by the hindgut microbiome further supports our hypothesis that copiotrophic taxa in the proximal GIT and oligotrophic taxa in the distal GIT could regulate glycan-degrading strategies from dietary to endogenous polysaccharides.

The hydrogenase and terminal reductase profiles also showed that different hydrogenogenic and hydrogenotrophic consortia drive region-specific fermentation strategies in the GIT ecosystem. A previous study reported that electron-bifurcating and fermentative hydrogenases account for most hydrogenase transcripts involved in H_2_ evolution among ruminal microorganisms [2]. Interestingly, our results showed that the hindgut microbiome of dairy cattle had a higher prevalence of groups A3, A2, A1, and B [FeFe]-hydrogenases, suggesting that microbial populations from the large intestine may contribute more to H_2_ production by fermentative and electron-bifurcating hydrogenases than the four-chambered stomach microbiome. This observation highlights that the lower-gut microbiome is far more important for dairy cattle than previously appreciated [3]. Of the hydrogenotrophic populations, we determined that the hindgut microbiome was a major agent of hydrogenotrophic methanogenesis and acetogenesis, whereas hydrogenotrophic fumarate reducers were mainly obtained from the four-chambered stomach. This result further suggests that research on the H_2_ cycling pathways employed by the GIT microbe, particularly the neglected lower-gut microbiome, is important for optimizing fermentation strategies by redirecting H_2_ flux from methanogens to alternative uptake pathways, and thus could improve the feed efficiency of dairy cattle.

Comparisons of the GIT microbial communities of the two dietary regimes further indicate that nutrient availability plays a critical role in altering microbial biodiversity and shaping community composition. Specifically, we found that the modified effects of diet were most apparent in the four-chambered stomach microbiome due to its preferential utilization toward dietary substrates. In contrast, the post-gastric GIT microbiome can consume mucin-derived glycans as carbon and energy sources, irrespective of the host diet [29], thus their colonizers are less susceptible to a host diet regime shift. Furthermore, we found that the loss of microbial richness during a grain-based diet did not appear to be completely stochastic with respect to microbial taxa in the four-chambered stomach. A grain-based diet depleted members of functionally cellulolytic bacteria in the four-chambered stomach, especially *Prevotella* sp. UBA3846, which possessed predicted cellulosomal enzymes and diverse hemicellulase PULs. A concomitant increase in starch-degrading bacteria in the four-chambered stomach after feeding on a grain-based diet also explains that substrate types selectively modify target populations. Therefore, in low-diversity communities of a grain-based diet, nutrient availability drives selective assembly processes associated with the reduction of unfavored functional taxa and the expansion of substrate-biased taxa, leading to lower-redundancy microbial niches. However, such landscapes may not be resilient to environmental change.

From the forage- to the grain-based regime, the amendment of polysaccharide degradation strategies selectively expanded the propionate-produced microbiota in the four-chambered stomach and small intestine, thereby creating a new ecological niche. During fermentation, hydrogen is a key junction that connects different functional groups in the rumen ecosystem [13]. Thus, competition for hydrogen can lead to the enrichment of one functional group over the other. When promoting fumarate reduction to propionate based on a grain-based regime in the four-chambered stomach, other hydrogenotrophic functional groups were changed, such as a reduction in acetogenesis. Shunting carbon toward propionate reduced nutrient availability for hydrogenotrophic acetogens in the proximal GIT, and this shift is consistent with the metabolic dynamics previously reported in rumen microbiomes [2, 30]. Altogether, different diet regimes can redirect H_2_ flux to remodel fermentation schemes and further studies are needed to determine how to optimize fermentation strategies to favor animal production.

## Conclusions

Collectively, our study deciphered the landscape of the region-specific trophic system and the diet-induced metabolic flexibility of the GIT microbiome in dairy cattle. We identified the highest diversity of the four-chambered stomach microbiome related to their recalcitrant polysaccharide substrates, underpinned by the prevalence of cellulosome-­producing and plant-degrading PUL-encoding consortia. In contrast, the prevalence of microbial glycan-degrading genes in the small intestine and host glycan-degrading genes in the large intestine was another trait of carbon source availability across GIT regions. The carbon and hydrogen metabolism profiles indicated that different functional groups had region-specific scatter across different GIT sites, including the prevalence of propionate-producing pathways and methylotrophic methanogenesis in the four-chambered stomach, a higher representation of acetogenesis and hydrogenotrophic methanogenesis in the post-gastric intestine. The shift from forage-based to grain-based regimes mainly modified specific populations of the four-chambered stomach in favor of starch degradation by narrowing down plant biomass hydrolysis. A grain-based diet further shunted carbon toward propionate and reduced substrate availability for hydrogenotrophic acetogens in the four-chambered stomach. Therefore, our research lays a foundation for developing an understanding of the GIT microbial organization and function of dairy cattle constrained by the deterministic drivers of the region and the modified effects of diet, which will contribute to improving animal health and milk production in dairy cattle.

## Methods

### Animals and experimental design

Twelve heathy Holstein cows (651 ± 54 kg; mid-lactation) with a mean milk yield of 17.4 ± 4.0 kg/day were housed in tie stalls for the 1-month experiment. Prior to the animal trial, all cows were fed a forage-based diet (F group) for 1 week. After the preparation period, six cows were randomly divided into the F group and continued to feed on a forage-based diet (Additional file 2: Table S26). The other six cows were shifted to feed on a grain-based diet (G group) with a forage/concentrate ratios of 4:6 on a day matter basis (Additional file 2: Table S26). The feeding trial lasted for 21 days, and the animals were fed twice per day (07:00 and 19:00) ad libitum.

### Sampling scheme

On the last day of the experiment, all dairy cattle were stunned and exsanguinated, and their internal organs were immediately dissected. The GIT (including the rumen, reticulum, omasum, abomasum, duodenum, jejunum, ileum, cecum, colon, and rectum) of each animal was separated and the lumen content of the GIT was homogenized separately. The pH of each GIT sample was immediately determined using a portable pH meter (catalog no. HI 9024C; HANNA Instruments, Woonsocket, RI, USA). One part of the sample was centrifuged at 12,000 rpm for 10 min and the supernatants were collected and stored at − 20 °C for analysis of volatile fatty acid concentrations by gas chromatography (GC-14B, Shimadzu, Japan) [31]. Next, 5 mL of homogenized content from each GIT region was sampled in triplicate and frozen in liquid nitrogen for DNA extraction.

### DNA extraction and metagenomic sequencing

Total DNA was extracted from all GIT samples (approximately 200 mg per sample) based on repeated bead-beating using a mini-bead beater (Biospec Products, Bartlesville, USA) [32]. The integrity of the extracted DNA was measured by electrophoresis on 0.8% agarose gels, and the quality and quantity were determined using a Nanodrop ND-1000 (Thermo Scientific, Wilmington, USA). Following the manufacturer’s instructions for the TruSeq DNA PCR-Free Library Preparation Kit (Illumina, San Diego, CA, USA), high-quality DNA from each sample was used to construct a metagenomic library with an insert size of 350 bp, and then sequenced on an Illumina NovaSeq platform.

### Construction of the GIT microbial gene catalog of dairy cattle

Trimmomatic [33] (v.0.33) was used to trim adapters from the Illumina data, and BWA-MEM [34] (v.0.7.17) was used to remove contaminated sequence data, including dairy cattle (*Bos taurus*, GCA_002263795.2), feed, and human sequences (*Homo sapiens*, GCA_000001405.28) (Additional file 1: Table S1). The reference genome sets of plants in feed included wheat (*Triticum aestivum*, GCA_002220415.3), medicago (*Medicago truncatula*, GCA_000219495.2), rice (*Oryza sativa*, GCF_000005425.2), maize (*Zea mays*, GCA_003185045.1 and GCA_000005005.6), and soybean (*Glycine max*, GCA_000004515.4). The high-quality reads from each sample were individually assembled using MEGAHIT [35] (v.1.1.1) and IDBA-UD [36] (v.1.1.3), and the contigs were combined using Minimus2 [37] (AMOS, v.3.1.0). BWA-ALN [38] (v.0.7.17) and SAMtools [39] (v.1.9) were used to reduce errors generated from the assembly process by mapping all reads back to the contigs, after which the single bases, insertions, and deletions were corrected. Next, the open reading frames (ORFs) were predicted using Prodigal [40] (v.2.6.3) with the parameter “-p meta,” and 153.4 million ORFs were generated with 29.9% completed ORFs. ORFs less than 100 bp were removed, and the remaining ORFs were clustered using CD-HIT [41] (v.4.8.1, parameter “-n 9 -g 1 -c 0.95 -G 0 -M 0 -d 0 -aS 0.9”). After removing redundant genes with ≥ 95% nucleotide sequence identity and ≥ 90% overlap [42], we obtained a non-redundant GIT microbial gene catalog of dairy cattle with 45.9 million genes.

### Taxonomic classification and functional annotation

The taxonomic and functional repertoires of the gene catalog were annotated using DIAMOND [43] (v.0.9.22), based on BLASTP searches against the NCBI-NR, eggNOG [44] (v.4.5.1), and KEGG [45] (v.90.0) databases. The highest scoring of the assigned orthologous group was considered as the annotated hit of each putatively encoded protein. The protein sequences were matched to the CAZyme database [46] based on a hidden Markov model for each protein using HMMER [47] (v.3.2.1). BWA-MEM [34] (v.0.7.17) was used to align high-quality reads from each sample against the gene catalog, and the abundance of each gene was calculated using the method of transcripts per million [48] (TPM, with an alignment length ≥ 50 bp and sequence identity > 95%). TPM is calculated as

$$\text{TPM}\,=\frac{{\text{N}}_\text{g}}{{\text{L}}_\text{g}}\times\frac1{\sum\text{j}\frac{{\text{N}}_\text{j}}{{\text{L}}_\text{j}}}\times10^6$$

where *N*_*g*_ is the read count, that is, the average number of reads mapped to the *g* gene; and *L*_*g*_ is the gene length, that is, the number of nucleotides in the *g* gene. The index *j* represents the set of all genes determined in the catalog, and *g* is an index indicating a particular gene [48]. The abundance of taxa, KOs, and CAZymes was summarized based on the abundance of annotated genes [42].

### Metagenomic binning

MaxBin [49] (v.2.2.4), MetaBAT2 [50] (v.2.11.1), and CONCOCT [51] (v.0.4.0) were used for metagenomic binning based on contigs from assemblies (> 1.5 kb) with default parameters. The bins generated from the three approaches were integrated using the DAS tool [52] (v.1.1.1). CheckM [53] (v.1.0.7) with a lineage_wf workflow was applied to estimate the completeness and contamination of all 23,356 bins, and the quality scores were defined as completeness − 5 × contamination [54]. After filtering with completeness ≥ 50% and contamination ≤ 10%, 3079 MAGs remained. These MAGs were dereplicated at an average nucleotide identity (ANI) cutoff using dRep [55] (v.2.5.4; parameter: -p 72 –ignoreGenomeQuality -pa 0.95 -sa 0.99 -cm larger), and 1904 SGBs were obtained. After filtering for completeness > 80%, contamination < 10%, and quality score > 50, 592 high-quality SGBs were obtained for further functional annotation. A total of 1904 ORFs were predicted using Prodigal [40] (v.2.6.3). The estimated genome size was corrected based on the completeness and contamination following the algorithm from Nayfach et al. [56], and the abundance of SGBs in each sample was assessed using metaWRAP [57] (v.1.3) with a “quant_bins” module based on the TPM calculation process.

### Phylogenetic, taxonomic, and functional analyses of genomes

A maximum-likelihood phylogenetic tree was constructed using PhyloPhlAn [58] (v.1.0) with 1904 SGBs, and visualized using Evolview [59] (v.3) and iTol [60] (v.4.3.1). All genomes were annotated using GTDB-Tk [61] (v.0.1.6) based on the Genome Taxonomy Database. The CAZyme families of the 592 high-quality genomes were annotated using HMMER [47] (v.3.2.1) based on a hidden Markov model. The PUL of high-quality SGBs was predicted by following the PULpy [62] (v.1.0) pipeline. The KOs of the high-quality SGBs were annotated using DIAMOND [43] (v.0.9.22) based on BLASTP searches against the KEGG [45] (v.90.0) databases. Protein sequences encoded by the 592 high-quality SGBs were also screened against HydDB [63] databases to identify the catalytic subunits of the three classes of hydrogenases ([NiFe]-, [FeFe]-, and **[**Fe]-hydrogenases) using BLASTP with an e-value threshold of 1e - 50, coverage values exceeding 90%, and identity values exceeding 50% [2].

### Statistical analysis

Ordination analysis of Bray–Curtis distances [64] between ten GIT regions or two dietary groups (F/G) based on the taxonomic profiles (species) at the gene and genome levels were defined, and the differences between groups were assessed using the PERMANOVA and ANOSIM test in the R vegan package [65] (v.2.5–6) with 9999 permutations, and then visualized using a PCoA plot. Alpha (richness index) and beta (Bray–Curtis dissimilarity) diversities were calculated based on the taxonomic profile using the R vegan package. To test two independent factors (region and diet) and the influence of their interaction on taxonomic and functional variance, we used the nonparametric Scheirer–Ray–Hare extension of the Kruskal–Wallis test. The taxonomic and functional matrices based on genes and SGBs were compared between different regions and diet groups using the Wilcoxon rank-sum test. The cutoff of the differential abundance of regions was set at *p* < 0.05 and that of diets at the genome level was log_2_^fold−change^ > 1 and *p* < 0.05. We used the *t*-test to statistically compare the fermentation parameters of the two diets.

## Supplementary Information

**Additional file 1:** **Table S1.** The assembly results of the 120 GIT content samples in dairy cattle. **Table S2.** Regional differences in microbial taxa at the phylum level among the GIT regions in dairy catlle fed forage-based (F) and grain-based (G) diets. **Table S3.** Regional differences in microbial taxa at the genus level among the GIT regions fed forage-based (F) and grain-based (G) diets. **Table S4.** The shared and specfic core metabolic pathways of the four-chambered stomach, small intestine, and large intestine in dairy cattle, respectively. **Table S5.** Comparing the abundance of Glycoside hydrolases (GH) and polysaccharide lyases (PL) familes of the microbiome across the GIT regions in dairy cattle fed forage-based (F) and grain-based (G) diets. **Table S6. **Comparison of levels of carbon metabolism modules KOs of the microbiome across the GIT regions in dairy cattle fed forage-based (F) and grain-based (G) diets. **Table S7.** Genomic statistics for 3079 MAGs (completeness ≥ 50% and contamination ≤ 10%) produced in this study. **Table S8.** Genomic statistics for 1904 non-redundant MAGs (completeness ≥ 50% and contamination ≤ 10%) produced in this study. **Table S9.** Regional differences in microbial populations at the genome level among the GIT regions in forage-based (F) and grain-based (G) diets. **Table S10.** The CAZyme-predicted proteins of 592 high-quality strain-level genome bins (SGBs; completeness > 80%, contamination < 10%, and quality score > 50). **Table S11.** The predicted polysaccharide utilization locus (PULs) of 202 high-quality SGBs among the GIT microbiota in dairy cattle. **Table S12.** The 213 high-quality SGBs encoding fermentative hydrogenases (H2-producing). **Table S13.** The 195 high-quality SGBs encoding [FeFe] Group A3 for bifurcating hydrogenases (bidirectional). **Table S15.** The different abundance in microbial taxa among the GIT regions between the forage-based (F) and grain-based (G) diets. **Table S16.** The different abundance of 1904 SGBs across the GIT regions between the forage-based (F) and grain-based (G) diets in dairy cattle. **Table S17.** The different abundance glycoside hydrolases (GH) and polysaccharide lyases (PL) familes of the microbiome across the GIT regions between the forage-based (F) and grain-based (G) diets in dairy cattle. **Table S18.** Comparison of the increased abundance of CAZymes across the GIT regions of dairy cattle fed a grain-based (G) diet. **Table S19.** Comparison of the decreased abundance of CAZymes across the GIT regions of dairy cattle fed a grain-based (G) diet. **Table S20.** The different abundance in carbon metabolism modules KOs of the microbiome across the GIT regions between the forage-based (F) and grain-based (G) diets in dairy cattle.

**Additional file 2:** **Fig. S1.** Pairwise comparison of (**a**) alpha diversity (Richness index) and (**b**) beta diversity (Bray-Curtis) among the GIT regions at the species levels of gene catalog in the forage-based (F) or grain-based (G) diets, respectively. Significance based on the relative index of each cohort according to the Wilcoxon rank-sum test. *p < 0.05, **p < 0.01, ***p < 0.001. FS, four-chambered stomach; SI, small intestine; LI, large intestine. **Fig. S2.** The dominant phyla in the GIT of the bacterial, archaeal, and eukaryotic communities in dairy cattle. Firm, Firmicutes; Bact, Bacteroidetes; Prot, Proteobacteria; Fibr, Fibrobacteres; Spir, Spirochaetes; Eury, Euryarchaeota; Nema, Nematoda; Chyt, Chytridiomycota. RUM, rumen; RET, reticulum; OMA, omasum; ABO, abomasum; DUO, duodenum; JEJ, jejunum; ILE, ileum; CEC, cecum; COL, colon; REC, rectum. Bacterial phyla are colored in blue, archaeal phyla are colored in green, and eukaryotic phyla are colored in red. **Fig. S3.** Pairwise comparison of the fermentation parameters among the FS, SI, and LI cohorts. Significance based on the relative index of each cohort according to the Wilcoxon rank-sum test. *p < 0.05, **p < 0.01, ***p < 0.001. FS, four-chambered stomach; SI, small intestine; LI, large intestine. **Fig. S4.** (**a**) Frequencies of phyla, classes, orders, families, and genera among the 1904 SGBs. The five most frequently observed taxa of each rank are shown in the legend, with the remainder grouped as ‘others’ and ‘unclassified’. (b) PCoA plot of 1904 SGBs among 10 GIT regions, with the color of circles indicating regions. RUM, rumen; RET, reticulum; OMA, omasum; ABO, abomasum; DUO, duodenum; JEJ, jejunum; ILE, ileum; CEC, cecum; COL, colon; REC, rectum. **Fig. S5. **Schematic representation of predicted PULs in targeted Vibrio cholerae RC9 spp. (SGB200, SGB9, SGB627, and SGB357). **Fig. S6. **Comparison of the fermentation parameters between the forage-based (F) and grain-based (G) diets from the proximal to distal GIT. Significance based on the relative index of each cohort according to the Wilcoxon rank-sum test. *p < 0.05, **p < 0.01, ***p < 0.001. RUM, rumen; RET, reticulum; OMA, omasum; ABO, abomasum; DUO, duodenum; JEJ, jejunum; ILE, ileum; CEC, cecum; COL, colon; REC, rectum. **Fig. S7.** The numbers of increased and decreased abundance of KOs between the forage-based (F) and grain-based (G) diets in each GIT region. RUM, rumen; RET, reticulum; OMA, omasum; ABO, abomasum; DUO, duodenum; JEJ, jejunum; ILE, ileum; CEC, cecum; COL, colon; REC, rectum. **Table S26.** Ingredients and nutritional compositions of the forage-based (F) and grain-based (G) diets.

**Additional file 3: Table S14.** The high-quality SGBs with sequences of hydrogenases and associated terminal reductases required for pathways of methanogenesis, acetogenesis, fumarate reduction, sulfidogenesis and nitrate.

**Additional file 4:** **Table S21.** The counts of CAZyme genes and KOs from carbon metabolism among the increased abundance of high-quality genomes in the grain-fed cows in the rumen.

**Additional file 5:** **Table S22.** The counts of CAZyme genes and KOs from carbon metabolism among the decreased abundance of high-quality genomes in the grain-fed cows in the rumen.

**Additional file 6:** **Table S23.** The counts of CAZyme genes and KOs from carbon metabolism among the decreased abundance of high-quality genomes in the grain-fed cows in the jejunum.

**Additional file 7:** **Table S24.** The counts of CAZyme genes and KOs from carbon metabolism among the increased abundance of high-quality genomes in the grain-fed cows in the cecum.

**Additional file 8:** **Table S25**. The shifted abundance of high-quality genomes with sequences of hydrogenases and associated terminal reductases required for pathways of fermentative hydrogenases, bifurcating hydrogenases, methanogenesis, fumarate reduction, sulfidogenesis and nitrate ammonification in the grain-fed animals.

## Data Availability

Raw sequence reads for all samples are available under European Nucleotide Archive (ENA) project PRJNA723218. All the 1904 SGBs utilized in this study have been deposited in Figshare (https://doi.org/10.6084/m9.figshare.14456637).
