# Supplementary material for: The gastrointestinal microbiome in dairy cattle is constrained by the deterministic driver of the region and the modified effect of diet
Source: Microbiome. 2023 Jan 20;11:10. doi: 10.1186/s40168-022-01453-2 (PMC9863278; doi:10.1186/s40168-022-01453-2)
Supplement: Supplementary file 3 — Additional file 2: Fig. S1. Pairwise comparison of (a) alpha diversity (Richness index) and (b) beta diversity (Bray-Curtis) among the GIT regions at the species levels of gene catalog in the forage-based (F) or grain-based (G) diets, respectively. Significance based on the relative index of each cohort according to the Wilcoxon rank-sum test. *p < 0.05, **p < 0.01, ***p < 0.001. FS, four-chambered stomach; SI, small intestine; LI, large intestine. Fig. S2. The dominant phyla in the GIT of the bacterial, archaeal, and eukaryotic communities in dairy cattle. Firm, Firmicutes; Bact, Bacteroidetes; Prot, Proteobacteria; Fibr, Fibrobacteres; Spir, Spirochaetes; Eury, Euryarchaeota; Nema, Nematoda; Chyt, Chytridiomycota. RUM, rumen; RET, reticulum; OMA, omasum; ABO, abomasum; DUO, duodenum; JEJ, jejunum; ILE, ileum; CEC, cecum; COL, colon; REC, rectum. Bacterial phyla are colored in blue, archaeal phyla are colored in green, and eukaryotic phyla are colored in red. Fig. S3. Pairwise comparison of the fermentation parameters among the FS, SI, and LI cohorts. Significance based on the relative index of each cohort according to the Wilcoxon rank-sum test. *p < 0.05, **p < 0.01, ***p < 0.001. FS, four-chambered stomach; SI, small intestine; LI, large intestine. Fig. S4. (a) Frequencies of phyla, classes, orders, families, and genera among the 1904 SGBs. The five most frequently observed taxa of each rank are shown in the legend, with the remainder grouped as ‘others’ and ‘unclassified’. (b) PCoA plot of 1904 SGBs among 10 GIT regions, with the color of circles indicating regions. RUM, rumen; RET, reticulum; OMA, omasum; ABO, abomasum; DUO, duodenum; JEJ, jejunum; ILE, ileum; CEC, cecum; COL, colon; REC, rectum. Fig. S5. Schematic representation of predicted PULs in targeted Vibrio cholerae RC9 spp. (SGB200, SGB9, SGB627, and SGB357). Fig. S6. Comparison of the fermentation parameters between the forage-based (F) and grain-based (G) diets from the proximal to distal GIT. Sign [file 40168_2022_1453_MOESM2_ESM.docx]

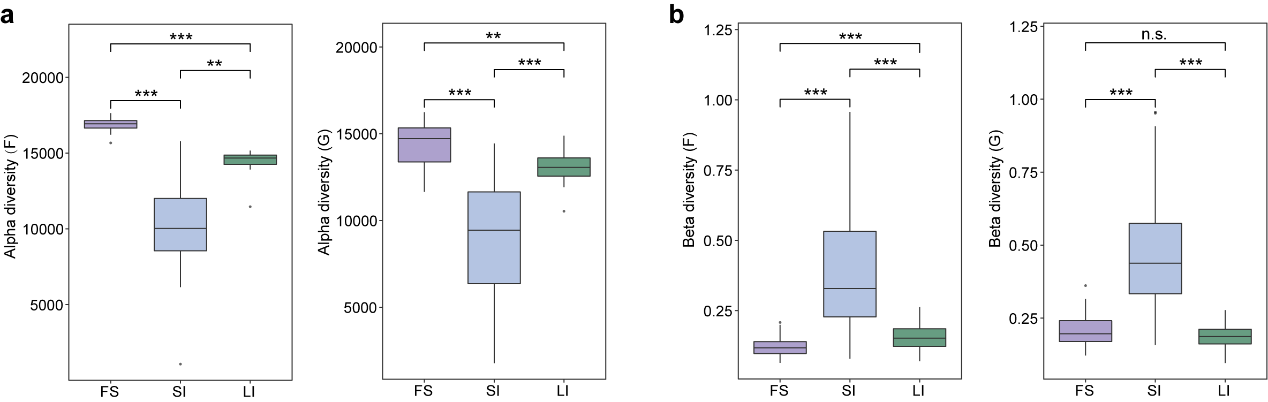


**Fig. S1** Pairwise comparison of (**a**) alpha diversity (Richness index) and (**b**) beta diversity (Bray-Curtis) among the GIT regions at the species levels of gene catalog in the forage-based (F) or grain-based (G) diets, respectively. Significance based on the relative index of each cohort according to the Wilcoxon rank-sum test. **p* < 0.05, ***p* < 0.01, ****p* < 0.001. FS, four-chambered stomach; SI, small intestine; LI, large intestine.


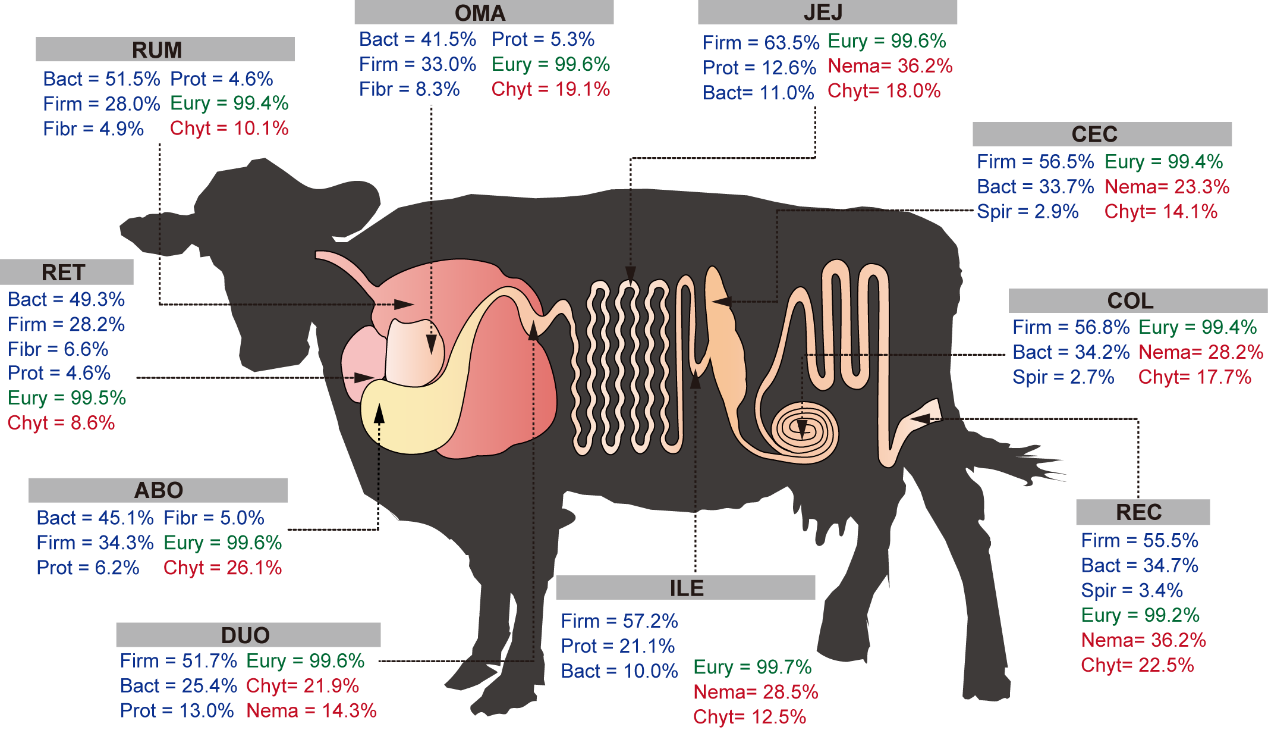


**Fig. S2** The dominant phyla in the GIT of the bacterial, archaeal, and eukaryotic communities in dairy cattle. Firm, Firmicutes; Bact, Bacteroidetes; Prot, Proteobacteria; Fibr, Fibrobacteres; Spir, Spirochaetes; Eury, Euryarchaeota; Nema, Nematoda; Chyt, Chytridiomycota. RUM, rumen; RET, reticulum; OMA, omasum; ABO, abomasum; DUO, duodenum; JEJ, jejunum; ILE, ileum; CEC, cecum; COL, colon; REC, rectum. Bacterial phyla are colored in blue, archaeal phyla are colored in green, and eukaryotic phyla are colored in red.

**
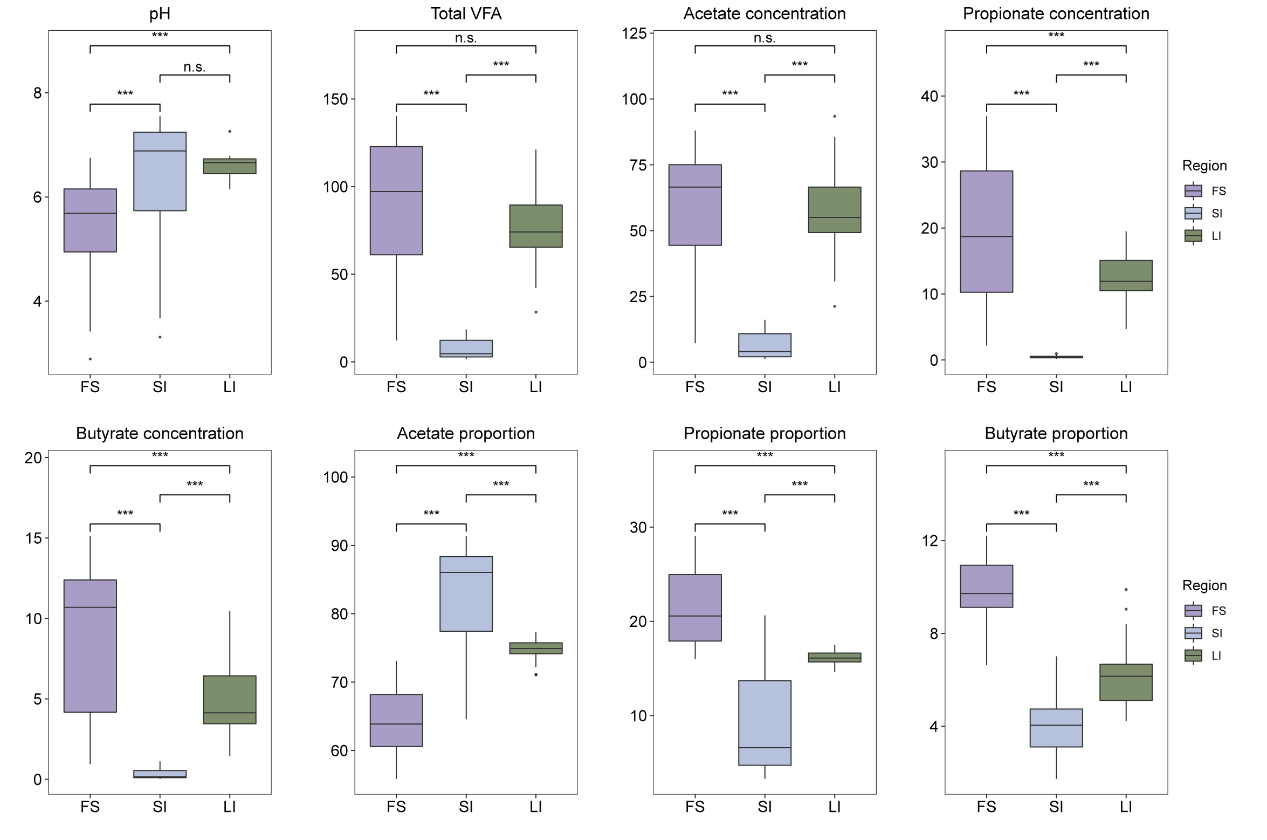
**

**Fig. S3** Pairwise comparison of the fermentation parameters among the FS, SI, and LI cohorts. Significance based on the relative index of each cohort according to the Wilcoxon rank-sum test. **p* < 0.05, ***p* < 0.01, ****p* < 0.001. FS, four-chambered stomach; SI, small intestine; LI, large intestine.

**
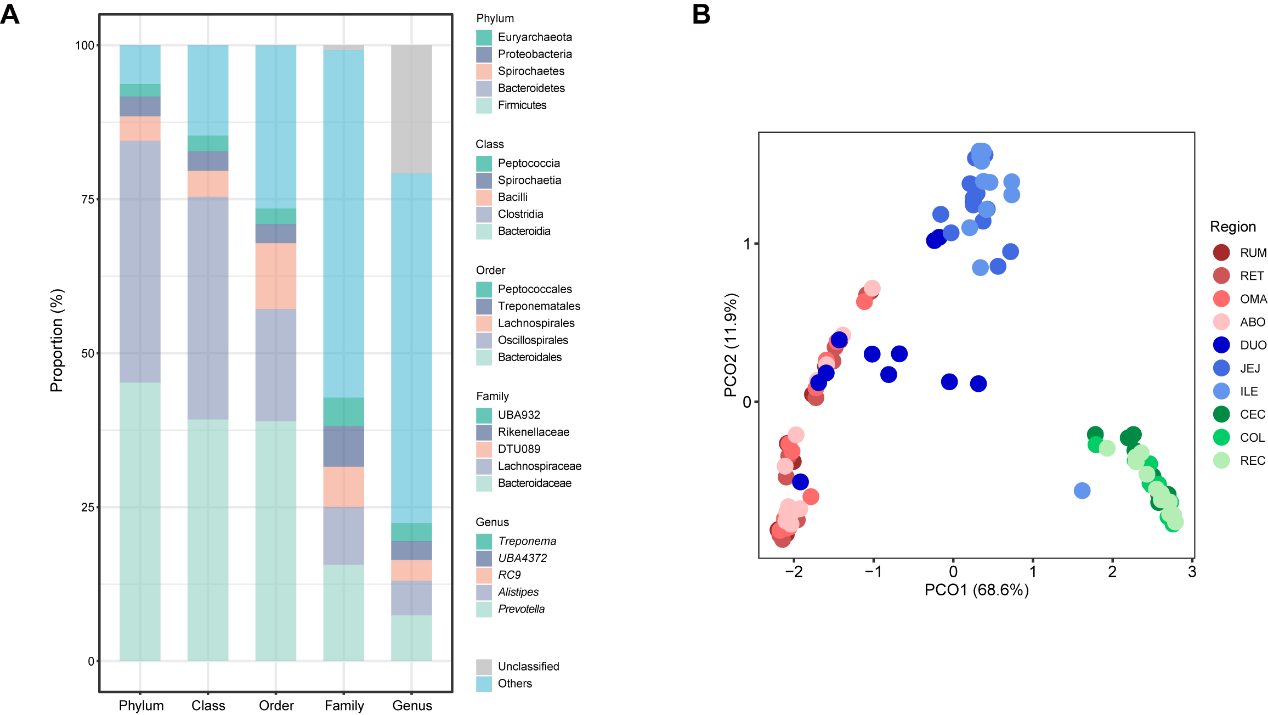
**

**Fig. S4** (**a**) Frequencies of phyla, classes, orders, families, and genera among the 1904 SGBs. The five most frequently observed taxa of each rank are shown in the legend, with the remainder grouped as ‘others’ and ‘unclassified’. (b) PCoA plot of 1904 SGBs among 10 GIT regions, with the color of circles indicating regions. RUM, rumen; RET, reticulum; OMA, omasum; ABO, abomasum; DUO, duodenum; JEJ, jejunum; ILE, ileum; CEC, cecum; COL, colon; REC, rectum.

**
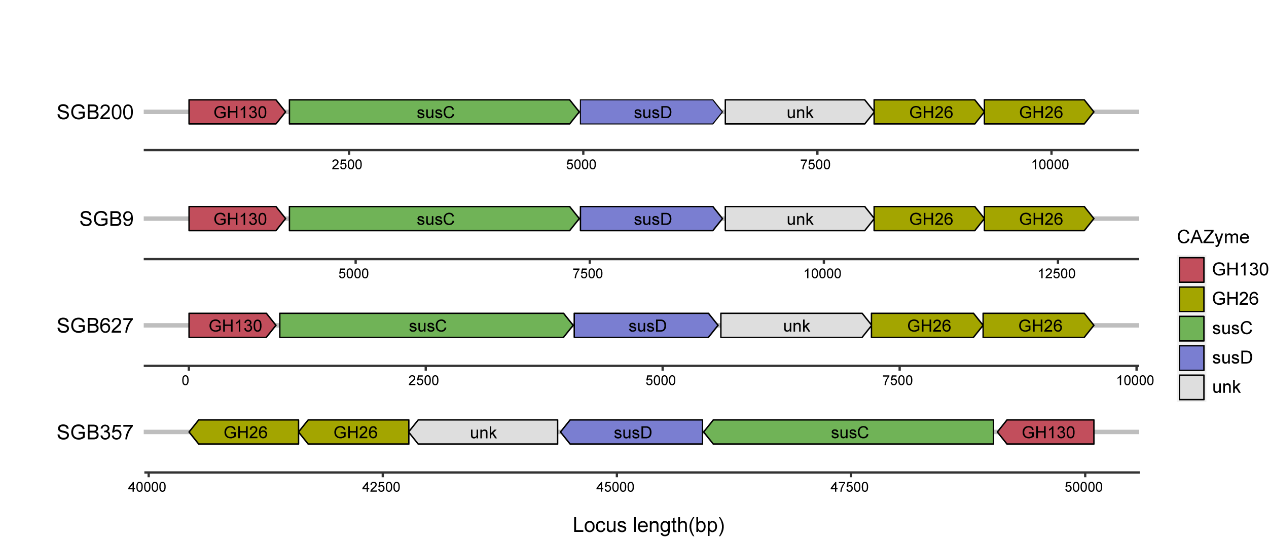
**

**Fig. S5** Schematic representation of predicted PULs in targeted Vibrio cholerae RC9 spp. (SGB200, SGB9, SGB627, and SGB357).


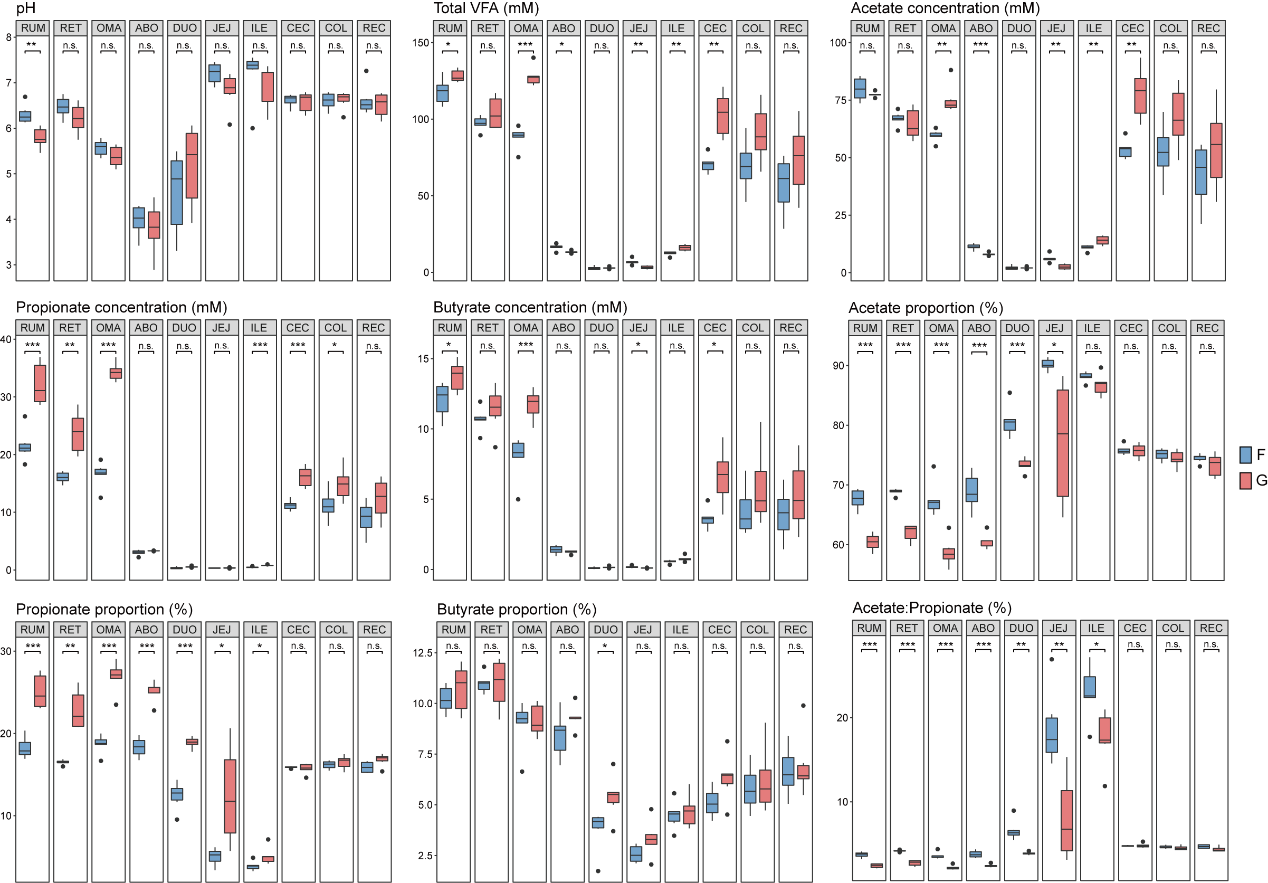


**Fig. S6** Comparison of the fermentation parameters between the forage-based (F) and grain-based (G) diets from the proximal to distal GIT. Significance based on the relative index of each cohort according to the Wilcoxon rank-sum test. **p* < 0.05, ***p* < 0.01, ****p* < 0.001. RUM, rumen; RET, reticulum; OMA, omasum; ABO, abomasum; DUO, duodenum; JEJ, jejunum; ILE, ileum; CEC, cecum; COL, colon; REC, rectum.


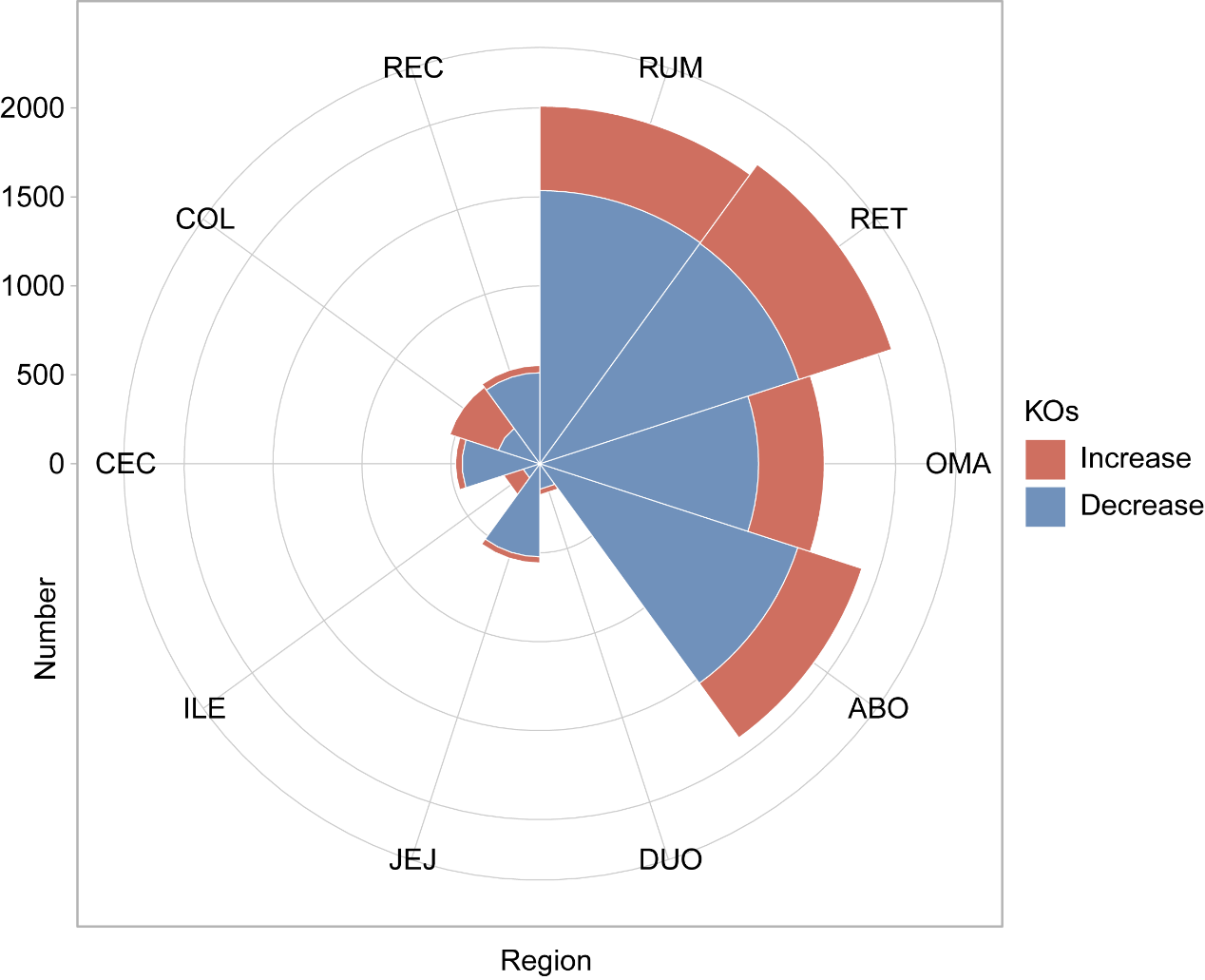


**Fig. S7** The numbers of increased and decreased abundance of KOs between the forage-based (F) and grain-based (G) diets in each GIT region. RUM, rumen; RET, reticulum; OMA, omasum; ABO, abomasum; DUO, duodenum; JEJ, jejunum; ILE, ileum; CEC, cecum; COL, colon; REC, rectum.

**Table S26.** Ingredients and nutritional compositions of the forage-based (F) and grain-based (G) diets.

| Item | F | G |
| --- | --- | --- |
| Ingredients, % of DM |  |  |
| Corn grain | 19.40 | 24.92 |
| Soybean | 13.50 | 13.48 |
| Barley | — | 12.00 |
| DDGS | 3.80 | 5.91 |
| CaCO_3_ | 0.80 | 1.48 |
| Ca(HCO₃)₂ | 1.10 | 0.92 |
| NaCl | 0.40 | 0.37 |
| Premix | 1.00 | 0.92 |
| Corn silage | 12.00 | 6.00 |
| American alfalfa hay | 24.00 | 17.00 |
| Australian oaten hay | 24.00 | 17.00 |
| Nutrients composition |  |  |
| DM, % | 46.77 | 48.03 |
| CP, % of DM | 16.16 | 16.12 |
| Crude fat, % of DM | 3.05 | 3.05 |
| NDF, % of DM | 36.14 | 29.92 |
| NFC, % of DM | 35.39 | 42.34 |
| Starch, % of DM | 17.96 | 27.82 |
| Ash, % of DM | 5.97 | 4.87 |
| Ca, % of DM | 1.14 | 1.18 |
| P, % of DM | 0.52 | 0.51 |
| NE_L_（Mcal/kg of DM） | 1.57 | 1.64 |
| NFC/NDF | 0.97 | 1.42 |

Premix contained the following ingredients per kilogram of diet: vitamin A, 22.5 KIU/kg; vitamin D3, 5.0 KIU/kg; vitamin E, 37.5 IU/kg; vitamin K3, 5.0 mg/kg; Mn, 63.5 mg/kg; Zn, 111.9 mg/kg; Cu, 25.6 mg/kg; and Fe, 159.3 mg/kg
